# Amplicon Sequencing of Single-Copy Protein-Coding Genes Reveals Accurate Diversity for Sequence-Discrete Microbiome Populations

**DOI:** 10.1128/spectrum.02105-21

**Published:** 2022-04-13

**Authors:** Chengfeng Yang, Qinzhi Su, Min Tang, Shiqi Luo, Hao Zheng, Xue Zhang, Xin Zhou

**Affiliations:** a College of Food Science and Nutritional Engineering, China Agricultural University, Beijing, China; b Department of Entomology, College of Plant Protection, China Agricultural University, Beijing, China; Howard University

**Keywords:** microbiota, SDP, quantification, 16S V4 region, metagenomics, *Gilliamella*, 16S

## Abstract

An in-depth understanding of microbial function and the division of ecological niches requires accurate delineation and identification of microbes at a fine taxonomic resolution. Microbial phylotypes are typically defined using a 97% small subunit (16S) rRNA threshold. However, increasing evidence has demonstrated the ubiquitous presence of taxonomic units of distinct functions within phylotypes. These so-called sequence-discrete populations (SDPs) have used to be mainly delineated by disjunct sequence similarity at the whole-genome level. However, gene markers that could accurately identify and quantify SDPs are lacking in microbial community studies. Here, we developed a pipeline to screen single-copy protein-coding genes that could accurately characterize SDP diversity via amplicon sequencing of microbial communities. Fifteen candidate marker genes were evaluated using three criteria (extent of sequence divergence, phylogenetic accuracy, and conservation of primer regions) and the selected genes were subject to test the efficiency in differentiating SDPs within *Gilliamella*, a core honeybee gut microbial phylotype, as a proof-of-concept. The results showed that the 16S V4 region failed to report accurate SDP diversities due to low taxonomic resolution and changing copy numbers. In contrast, the single-copy genes recommended by our pipeline were able to successfully quantify *Gilliamella* SDPs for both mock samples and honeybee guts, with results highly consistent with those of metagenomics. The pipeline developed in this study is expected to identify single-copy protein coding genes capable of accurately quantifying diverse bacterial communities at the SDP level.

**IMPORTANCE** Microbial communities can be distinguished by discrete genetic and ecological characteristics. These sequence-discrete populations are foundational for investigating the composition and functional structures of microbial communities at high resolution. In this study, we screened for reliable single-copy protein-coding marker genes to identify sequence-discrete populations through our pipeline. Using marker gene amplicon sequencing, we could accurately and efficiently delineate the population diversity in microbial communities. These results suggest that single copy protein-coding genes can be an accurate, quantitative, and economical alternative for characterizing population diversity. Moreover, the feasibility of a gene as marker for any bacterial population identification can be quickly evaluated by the pipeline proposed here.

## INTRODUCTION

Accurate identification of distinct functional units in natural bacterial communities is crucial in understanding their ecological roles, interactions within the network, as well as the fine-scale composition and dynamic changes within the whole community. As a rule of thumb, a bacterial phylotype is often defined by grouping strains that share a sequence identify greater than 97% for a selected fragment of the small subunit (16S) rRNA gene (1). However, increasing evidence has indicated that a bacterial phylotype may contain multiple finer lineages, each showing distinct biological traits. For example, closely related enterotoxigenic Escherichia coli (ETEC) isolates form discrete lineages with consistently definable variations in virulence profiles (2). Such intraphylotype lineages could be delineated based on divergence in genomic sequences and phylogenetic inferences. These finer subdivisions of phylotypes are called sequence-discrete populations (SDPs), which typified by genetic and genealogical discontinuity from the rest of the community and are delineated by overall sequence divergence at the whole-genome level (3–5). A broad comparison of 90,000 bacterial genomic sequences, with a close examination of pairwise genomic similarities in natural bacterial communities, has proved the pervasive discontinuity in genetic similarity below and above SDPs (3). Bacteria in the same SDP normally show less than ca. 5% variation in whole-genome sequences. This genetic divergence is much less than those among strains of the same phylotype (ca. 30%) (6). With respect to habitats, specific SDPs are likely ubiquitous in various environments, such as human and animal guts (5, 7, 8), freshwater (9), ocean (10) and soil (11). Based on large-scale whole-genome and metagenomics investigations, these genetically and ecologically discernible SDPs have been identified within multiple phylotypes, e.g., for intestinal Prevotella copri (8, 12) and Eubacterium rectale (13), four to five geographically stratified SDPs consist in human (or mouse) spanning geography and lifestyle. Therefore, SDPs are probably better than phylotypes, as taxonomic units that represent functional entities in bacterial communities, which are likely shaped by ecological pressure and evolutionary selection. As such, SDPs are important units of microbial diversity and should be considered baseline information for investing crucial questions, such as “how do bacterial populations interact and evolve within communities?” (4).

Despite the essential nature of accurate SDP identification, a rapid and accurate method that can trace SDP boundaries is still lacking, especially with regard to the selection of proper markers for evaluating sequence divergence. It is obvious that genetic divergence among bacterial strains is dependent on which genes are compared. We now understand that the commonly used 16S gene cannot generally provide sufficient resolution to characterize SDP diversity (14, 15). For example, in cases where the SDPs show an ∼5–10% genome-wide divergence, they varied mostly merely < 1% in the 16S sequences (16). Moreover, the copy number of the 16S gene may vary significantly among phylotypes or even among strains of the same phylotype, making quantitative characterization of bacterial community a challenging, if not impossible, task (17, 18). The 16S was selected for phylotype delineation years ago because it has conserved primer sites that flank relatively variable regions that made it easy to sequence with Sanger technology. Currently, much effort has been put into developing genes or gene segments that can be easily sequenced, and that vary enough to serve as practical proxies for SDP delineation (19–21). However, a systematic evaluation of the validity and performance of such genes in SDP delineation, which includes the rapidly increasing but heterogeneously sampled database, has not been carried out.

Fortunately, recent developments in microbial genomics show a promising solution to complement the coverage of bacterial genomes. The number of sequenced genomes of various bacterial lineages has been growing rapidly. For example, the Genomes OnLine Database (GOLD) now contains 437,099 bacterial genomes, the majority of which (397,945) are uncultured, representing host-associated, environmental and engineered ecosystems (22). The ever-growing bacterial genome data set offers a great opportunity to screen phylogenetically informative genes that show good performance in taxonomic delineation, including those capable of quantitatively characterizing bacterial communities at the SDP level (23, 24). For instance, Wu and colleagues identified 114 PhyEco universal markers for all bacteria (25). From these universal markers, 15 single-copy protein-coding genes were successfully applied in estimating species abundances using shotgun metagenomic data (26). On the other hand, growing numbers of genomes and metagenomes produced for particular bacterial communities or taxonomic groups allow for comprehensive characterization of SDP diversity within focal environments and bacterial groups. Taking social bee gut microbiota as an example, diverse strains derived from the major honeybee hosts have been isolated and deep-sequenced (27), including well-covered SDPs of nearly all core gut bacterial phylotypes (5, 28, 29). Thus, the relatively complete genome data set provides a genome-wide-based gold standard for defining SDPs for the honeybee core bacteria.

Honeybees are important pollinators and play vital roles in the sustainability of ecosystem and agriculture (30). Honeybee has a simple and specialized gut microbial community, consisting of 5–9 core bacterial genera (31, 32), which benefit the host in multiple aspects, including diet digestion (33, 34), nutrient provision (35), pathogen resistance (36), immune modulation (37) and endocrine signaling (35). Despite of its relatively simple diversity at the generic level, the gut bacterial community of the honeybee contains extensive genetic divergences, where high degrees of gene content diversity have been described within each phylotype (38–40). Strains belonging to the same phylotype form distinct phylogenetic clusters according to host species, e.g., *Lactobacillus* Firm5 (a dominant gut symbiont of honeybees) strains isolated from honeybees and bumble bees are grouped into 4 and 2 SDPs, respectively, showing distinct host specificity (38, 40). Shotgun metagenomics further revealed that SDPs generally co-occurred in individual honeybees with a relatively stable abundance (5). As gene repertoires can differ markedly among SDPs (38, 40), SDP profiling is essential for resolving the fine-scale diversity and the organization of ecological function in bee gut microbial community, as well as for understanding their impacts on the hosts.

**FIG 1:**
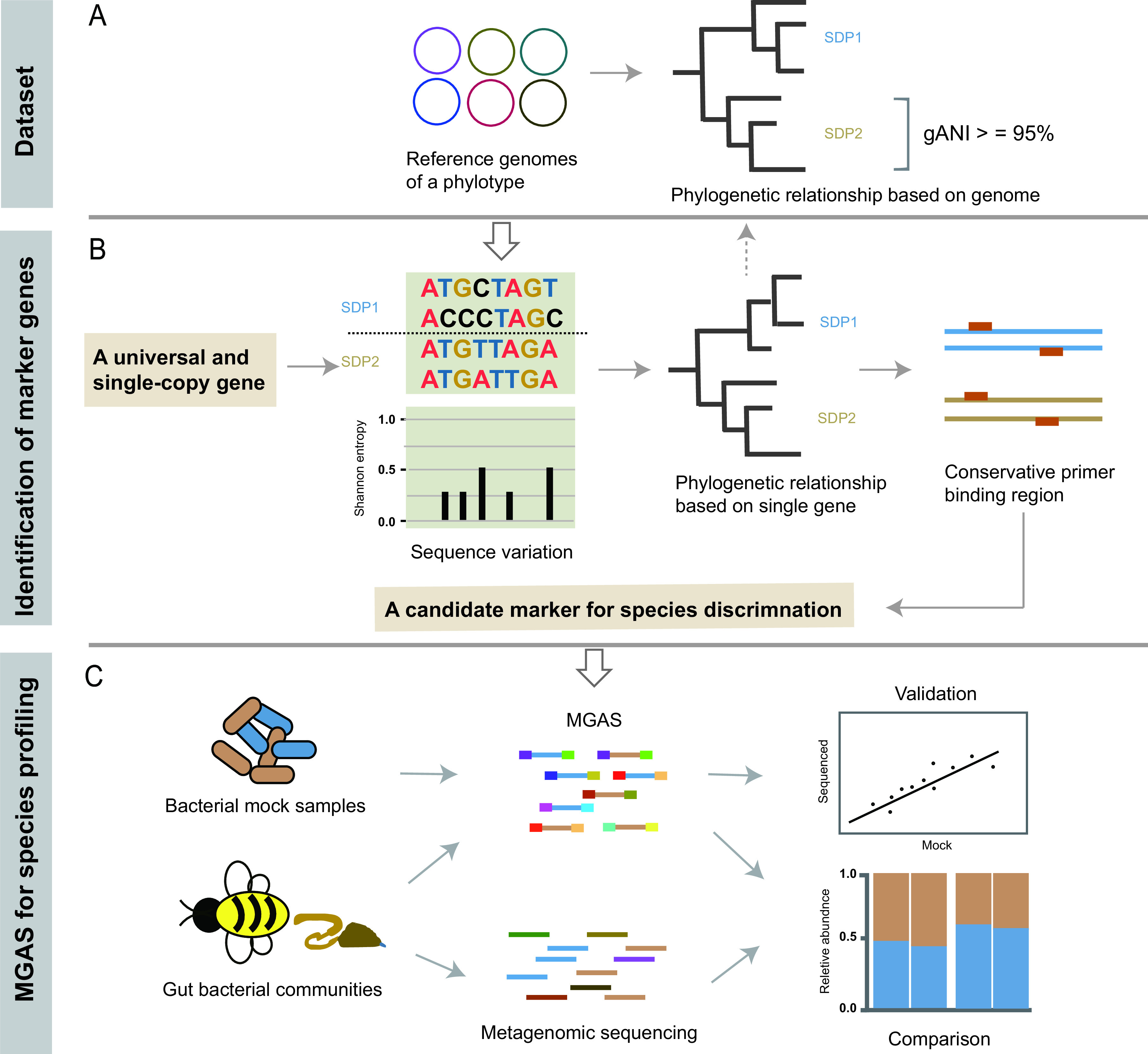
Screening marker genes suitable for SDP discrimination and quantification. (A) SDPs are identified for gut bacterial phylotypes based on phylogenetic relationships and genome-wide pairwise average nucleotide identities (gANI). (B) A candidate marker gene for SDP discrimination is selected from a set of universal and single-copy genes based on sequence variation, phylogenetic relationship, and well-conserved regions for primer design. (C) The performance of marker gene amplicon sequencing (MGAS) on SDP identification and quantification is validated and compared as characterized using the mock samples and gut communities.

In the present study, we developed a pipeline to screen potential marker genes capable of accurate identification and quantification of SDP diversity. Here, we have comprehensively collected core bacterial strains from the Asian honeybee Apis cerana across China. Among these core bacterial phylotypes, all known SDPs are well represented by numerous strains, and at least one strain has been genome-sequenced for each SDP. Therefore, we used *Gilliamella* as a proof of concept to examine the efficacy of the selected marker gene. In particular, we applied the available genome sequences as a leverage to delineate *Gilliamella* SDPs, which serves as the reference for marker evaluation. We further screened from a 15 single-copy protein-coding gene set leveraged from MIDAS software (26), which had been broadly applied for identification and quantification bacterial species from shotgun metagenomic data, to identify candidate marker genes capable of differentiating the defined *Gilliamella* SDPs. Important characteristics such as the level of sequence divergence, phylogenetic robustness, and the presence of conservative primer regions, are considered in marker gene screening. Finally, we applied the candidate markers in amplicon sequencing of both bacterial mock samples and real honeybee guts to verify their efficiency in SDP profiling (Fig. 1). The markers we identified could accurately, consistently and quantitatively capture SDP diversity.

## RESULTS

### A comprehensive genome reference database for honeybee gut bacteria.

A comprehensive genome reference database was constructed for honeybee gut bacteria (Table S1). A total of 242 genomes were included, covering 103 isolates from *A. cerana* and 139 from *A. mellifera*. Based on whole-genome sequence identity (gANI ≥ 95%), sequence discrete populations (SDPs) within each phylotype were identified. In line with previous studies, genomes associated with *A. cerana* and *A. mellifera* clustered into different SDPs (29, 39). For phylotypes associated with *A. cerana*, 5 SDPs were identified for *Gilliamella* (Gillia, n = 65), 2 for *Bifidobacterium* (Bifido, n = 9), 1 for *Lactobacillus* Firm5 (Firm5, n = 6), 1 for *Apibacter* (Apib, n = 16) and 2 for *Snodgrassella* (Snod, n = 7). For those associated with *A. mellifera*, 6 SDPs were identified for Gillia (n = 65), 9 for Bifido (n = 19), 2 for *Lactobacillus* Firm4 (Firm4, n = 2), 6 for Firm5 (n = 18) and 2 for Snod (n = 35) (Table S1). These SDPs delineated based on genome sequences were used as references for subsequent taxonomic assignments using 16S rRNA gene, marker gene, or metagenome-based SDP identifications.

### Single-copy marker genes showed higher sequence variations at the SDP level than the 16S gene.

**FIG 2:**
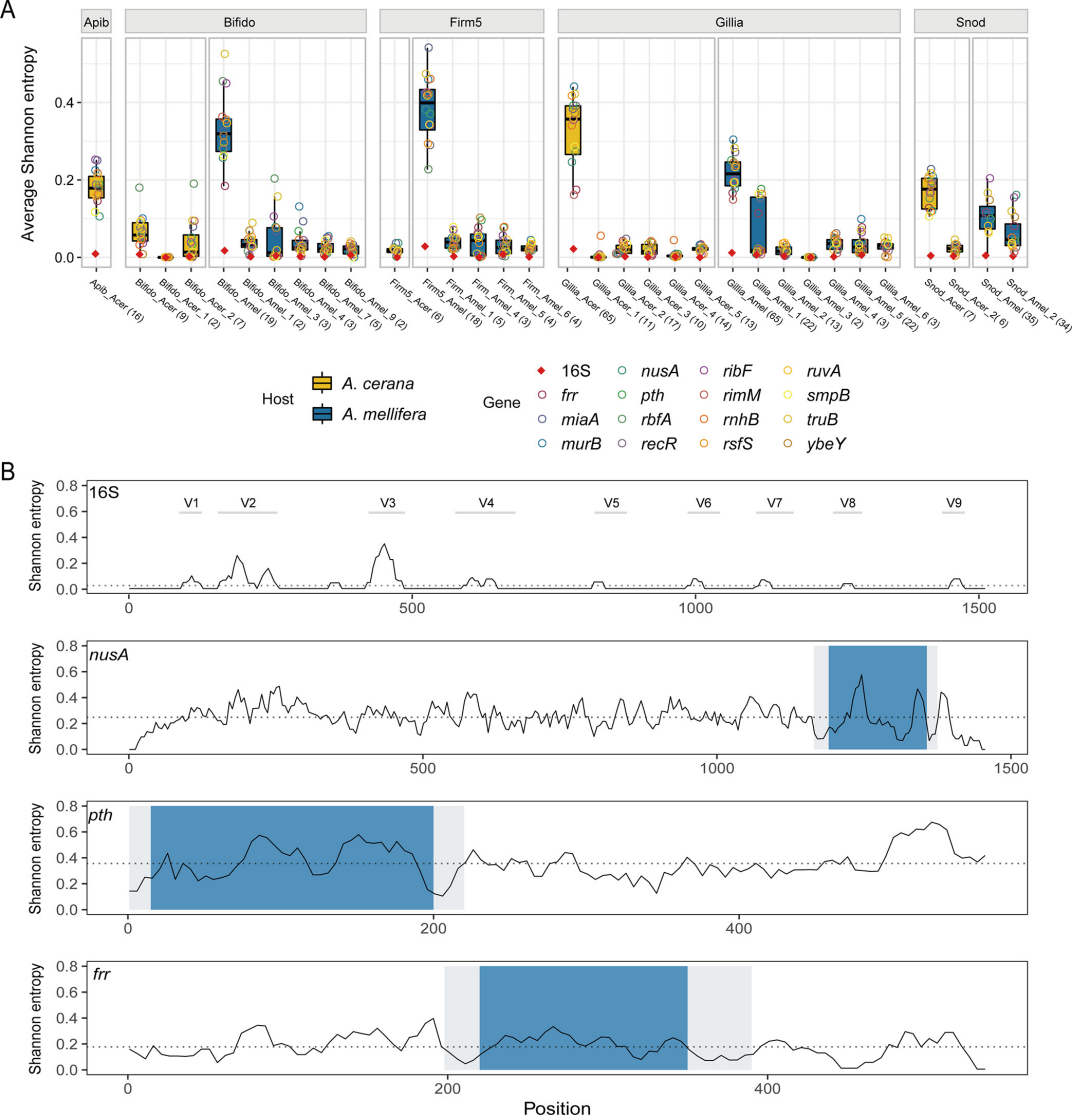
Marker genes are highly variable among SDPs. (A) Average Shannon entropy of the 15 marker genes and the 16S gene at both phylotype and SDP levels of honeybee gut bacteria. Numbers in brackets for each of the SDP groups indicate the number of strains examined for that specific group. (B) The Shannon entropy across 16S and candidate marker genes of all *A. cerana Gilliamella*. The Shannon entropy value is subsequently averaged by a 20-bp slide-window at a 5-bp step. Gray shadows depict conserved regions optimal for primer-binding sites and blue shadows are considered hypervariable regions in this study. Dash lines represent the mean Shannon entropy values cross all sequences. Gray lines depict the classic variable regions of the 16S gene. Apib: *Apibacter*; Bifido: *Bifidobacterium*; Firm5: *Lactobacillus* Firm5; Gillia: *Gilliamella*; Snod: Snodgrassella alvi.

Sufficient sequence variation is crucial for high resolution discrimination of bacterial SDPs. Here, we compared the average Shannon entropy (ASE) between the whole-16S and the 15 single-copy marker genes. Our results clearly showed that the marker genes had much higher ASEs at both phylotype and SDP levels compared to those of the 16S (Fig. 2A). The regional difference in the variation levels between 16S rRNA and selected marker genes was also compared along the full gene length. A slide-window (20 bp) SE analysis showed that several spikes of variable regions were identified along the 16S gene, with the highest variable region corresponding to part of the classic V3 region, mirroring results reported in a previous study, where the V3-V4 primers performed well in profiling bee-associated microbial communities (41). However, the taxonomically informative region of V3 is confined to its hypervariable fragments and is short in length (∼90 bp), which restrains its potential resolution power. Comparatively, marker genes are typically several folds longer in length, with informative sites evenly distributed along the gene, e.g., *nusA*, *pth*, and *frr* (Fig. 2B; Fig. S1).

Because phylogenetic placement of the query sequence is a critical step in our SDP identification method, each marker gene will need to first produce a “correct” phylogeny for the phylotype in question. Therefore, we further examined whether each of the 15 marker genes could produce the same SDP phylogeny as inferred from whole-genome sequences of *Gilliamella*. Here, the tree based on all 65 *A. cerana Gilliamella* genomes was used as the gold standard. The results showed that all 15 marker genes but *rnhB* reconstructed the SDP phylogeny, with all strains assigned to corresponding SDPs (Fig. S2). On the *rnhB* gene tree, two *Gilliamella* genomes were misplaced from SDP Acer_Gillia_4 to Acer_Gillia_2, which was likely due to a higher sequence similarity between these two SDPs at a value of 90.93% ± 0.18 SD comparing to that between other SDPs (79.98% ± 1.89 SD). Therefore, *rnhB* was subsequently excluded from further screening.

For the 14 remaining marker genes, we further explored for regions that were suitable for amplicon sequencing, based on the presence of conserved primer regions flanking the hyper-variable region. The *rimM* gene lacked hyper variable regions across the full gene length (Fig. S1), while some other genes (*murB, recR, miaA, rbfA, ribF, ruvA, rsfS*, and *yebY*) did not demonstrate promising conserved regions for primer design. These genes were then excluded from the candidate gene pool. The 5 remaining candidates (*frr*, *nusA*, *pth, truB*, and *smpB*) all had a hyper-variable region of ∼200-550 bp that was flanked by conservative primer regions. Among them, *frr*, *nusA* and *pth* produced an amplicon of ∼200 bp (Fig. 2B), which could be thoroughly sequenced with most current shotgun sequencing methods (e.g., PE100 or PE150). These 3 genes were then chosen for the final test for their performance in SDP discrimination in both identity and quantity, using *Gilliamella* mock samples and real honeybee guts.

### Marker gene amplicon sequencing (MGAS) showed high accuracy, sensitivity and repeatability in SDP profiling of mock samples.

Mock samples contained varied proportions of the representative strain cultures of the 5 *Gilliamella* SDPs. These samples were extracted for DNA and amplified for the hyper-variable regions of the 3 candidate marker genes (*frr*, *nusA*, and *pth*). Twenty-four barcoded amplicons were pooled and shotgun sequenced for ca. 1 Gb data (ca. 2.5 million reads). Each mock sample was sequenced three times. An average of 73,462, 86,467, and 113,498 reads per sample was generated for *frr*, *nusA* and *pth*, respectively.

**FIG 3:**
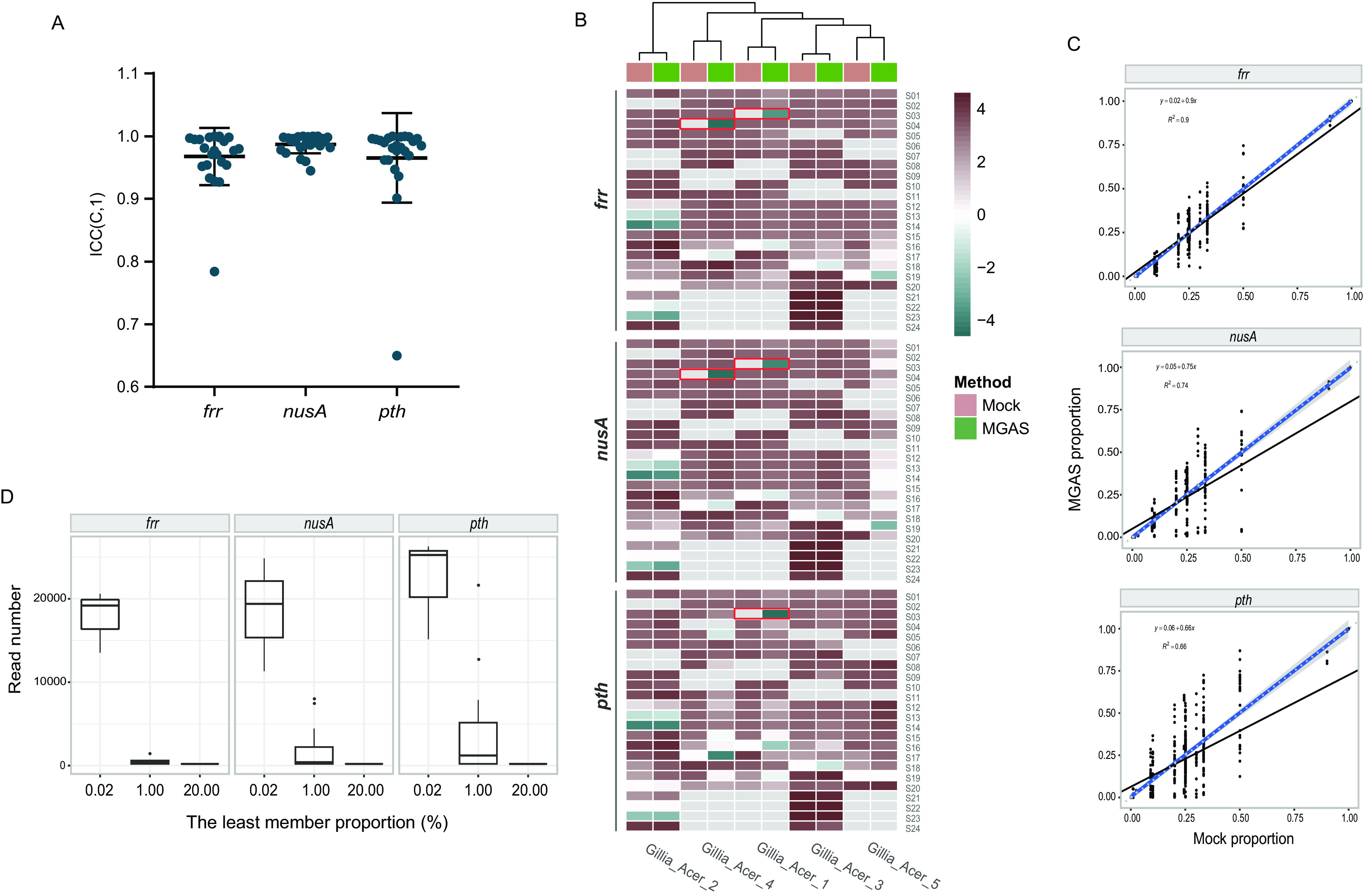
MGAS accurately identifies *A. cerana Gilliamella* SDPs. (A) Intraclass correlation coefficient (ICC) of relative abundance among the three replicates of MGAS samples. The ICC is calculated using the two-way mixed effects model with consistency (C) as the relationship among replicates, and single (1) result as the unit of measurement, i.e., ICC(C, 1). (B) Relative SDP abundances in mock samples revealed by marker gene sequencing. The results shown in the heatmap are the logarithms of the relative abundances of the five representative strains of the five SDPs of *A. cerana Gilliamella*. Gray box indicates a relative abundance at zero. False positive results are framed in red. (C) Spearman correlation of SDP abundances in *A. cerana Gilliamella* communities revealed by sequencing against mock samples. *P* < 2.2e-16. The black line presents the linear regression of the MGAS results against SDP abundances in mock samples. The blue solid and gray dashed lines represent a 1: 1 line and the fitted exponential regression (with 95% confidence interval shown in gray shade), respectively. (D) Minimum read numbers required for detecting members at low abundances.

The results of MGAS showed a high level of repeatability across the three replicates, where the average ICC(C,1) > 0.9, except for *pth*, which had an ICC(C,1) of 0.752 among samples with equal proportion of bacterial DNA (Fig. 3A; Fig. S4C). With regard to detection accuracy, MGAS correctly detected all bacterial members present in 22/24 samples, while two samples (S03 and S04) showed false-positive results, which was probably derived from sample contamination or sequencing error (Fig. 3B). Because the sensitivity of amplicon sequencing was affected by sequencing depth, we calculated the minimum read numbers required to detect members at low abundances, using rarefaction curves (Fig. S5). The results suggested that strains with a relative abundance of 1% could be detected by a minimum of ca. 1,123, 2,953, and 5,034 reads for *frr*, *nusA*, and *pth* (equivalent to 0.49, 1.29, and 2.44 Mb data per sample), respectively. Accordingly, lower abundance would require deeper sequencing. At a relative abundance of 0.02%, approximately 17,778, 18,518, and 22,222 reads (7.75, 8.07, and 10.76 Mb data) were required for *frr*, *nusA*, and *pth*, respectively (Fig. 3D; Fig. S5). The sequencing depth was generally sufficient for SDP detection in our study. Among the 216 sequenced samples, only two samples were sequenced with only 963 (*frr*) and 2,348 (*pth*) reads, respectively, and failed in identifying corresponding SDP members at the lowest proportions (1% and 0.1%, respectively) due to insufficient sequencing depth.

All three markers performed well for the DNA mixture with the average R^2^ values of 0.99, 0.91 and 0.99, for *frr*, *nusA*, and *pth*, respectively (Fig. S4B). In addition, mock samples collected from bacterial culture mixtures were also sequenced. The relative abundances evaluated by frr amplicon reads were highly congruent with the corresponding proportions of the mock samples, with the average R^2^ values of 0.91, while *nusA* and *pth* showed relatively low fidelities with R^2^ values of 0.74 and 0.66, respectively (*P* < 2.2e-16, Fig. 3C). The reduced accuracy in bacterial culture mixtures was likely attributed to artifacts associated with the sample homogenizing procedure. Overall, the MGAS method showed high levels of accuracy, sensitivity and repeatability in characterizing SDP compositions, in both taxonomic identity and relative abundance.

### MGAS performed equally well as metagenomics in characterizing honeybee gut SDP diversity.

**FIG 4:**
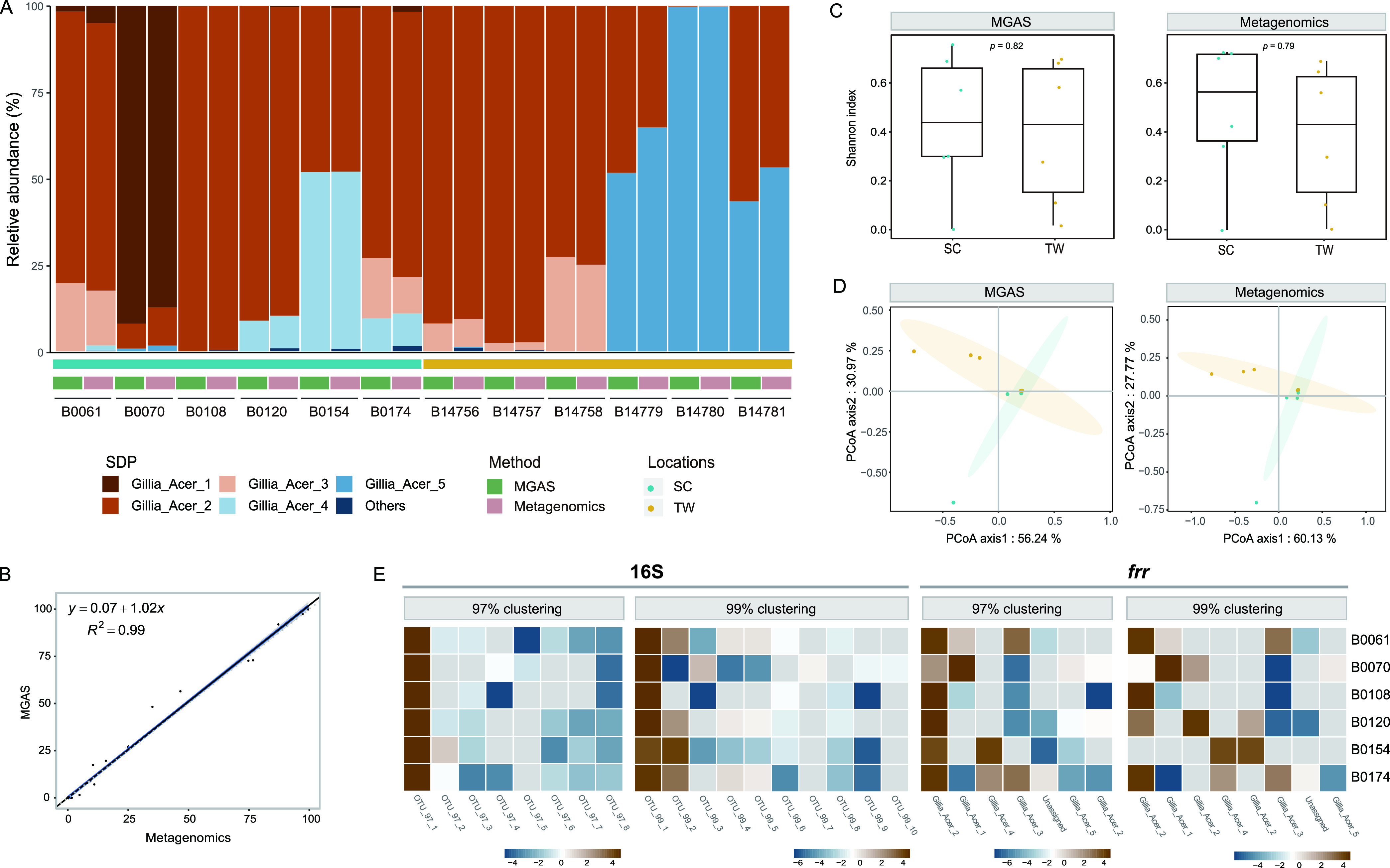
MGAS shows high congruence to metagenomics sequencing at SDP-level analysis. (A) Relative abundances of *Gilliamella* SDPs revealed by MGAS (*frr*) and metagenomics sequencing of *A. cerana* gut communities. (B) Spearman correlation coefficient between MGAS and metagenomics results, with R^2^ = 0.99, *P* < 2.2e-16. The black line presents the linear regression of the MGAS results in SDP abundances against those of metagenomics. The blue solid and gray dashed lines represent a 1: 1 line and the fitted exponential regression (with 95% confidence interval shown in gray shade), respectively. (C) Shannon diversity index of SDP frequencies for bee guts from two locations calculated by MGAS (left panel) and metagenomics sequencing (right panel). The two methods showed no significant difference, with the *P*-value of 0.70 and 0.82 in SC and TW, respectively, by Wilcoxon rank-sum test. (D) Principal coordinate analysis (PCoA) based on Bray-Curtis dissimilarity of SDP compositions of honey bee workers from Sichuan and Taiwan using MGAS (left panel, Adonis PERMANOVA, R^2^ = 0.056, *P* = 0.204) and metagenomics sequencing (right panel, Adonis PERMANOVA, R^2^ = 0.096, *P* = 0.134). Each point represents the value for an individual bee, with the color showing its collection location (Sichuan or Taiwan). Note that samples B0108, B0120, B14756, B14757, and B14758had similar *Gilliamella* SDP compositions, therefore overlapped in the figure. The shaded ellipses represent 95% confidence intervals on the ordination. (E) Relative abundances of *Gilliamella* OTUs in the gut microbiota of *A. cerana* assigned by clustering at 97% or 99% thresholds for 16S V4 and *frr*. The result shown in the heatmap are the logarithms of the relative abundances of the OTUs or five SDPs. Individual bees are marked to right of each row. Gray box indicates a relative abundance at zero.

To examine the performance of the MGAS method in characterizing honeybee gut microbiota, we used *frr* (Fig. 4) and *pth* (Fig. S6) genes to calculate *Gilliamella* SDP diversities for the 12 *A. cerana* workers from Sichuan and Taiwan, China. The MGAS was able to assign strains to the correct SDP at accurate abundance for real gut samples, with results were highly congruent with those from metagenomics sequencing (with R^2^ = 0.99 for *frr* and 0.97 for *pth*, *P* < 2.2e-16, Fig. 4B; Fig. S6B). Both results revealed that most individual bees were dominated by two or three *Gilliamella* SDPs, yet with significant variations in dominant members and compositions among individuals and across geographical locations (Fig. 4A). Gillia_Acer_2 was the dominant SDP in most of the sequenced bees, which was found in 11 out of the 12 samples, with 10 bearing relative abundances of 48.06–98.37% (Fig. 4A). Both methods showed congruent results in alpha diversity (*P* = 0.82 and 0.79 for MGAS and metagenomics sequencing, respectively, Wilcoxon rank-sum test, Fig. 4C). At the beta diversity level, the principal coordinate analysis (PCoA) based on Bray-Curtis dissimilarity revealed that the gut bacterial communities from bees of Sichuan and Taiwan formed two distinct clusters, which separated along the first axis (Fig. 4D). This result was again consistent between the MGAS and metagenomic methods (Adonis PERMANOVA, R^2^ = 0.056, *P* = 0.204 for MGAS and R^2^ = 0.096, *P* = 0.134 for metagenomics). Thus, the performance of SDP profiling using MGAS was parallel to the metagenomic gold standard in microbial community studies.

The 16S V4 region was also used to determine the *Gilliamella* SDP compositions for the 6 bee gut samples from Sichuan. We applied operational taxonomic unit (OTU) clustering based on sequence similarity at 97% and 99% identity thresholds, which are commonly adopted for surveying phylotype and intraphylotype microbial diversities, respectively (14, 42), to assess the efficacy of 16S V4 region in SDP profiling. 16S V4 amplicon sequencing resulted in 8 and 10 OTUs at 97% and 99% thresholds, respectively, with a frequency cut off at > 100. The identified OTU numbers differed from those of the MGAS results at the same sequence similarity thresholds (Fig. 4E). Alarmingly, 16S V4 amplicons failed to assign OTUs to the correct SDPs via blast. And the relative OTU proportions revealed by 16S V4 region disagreed with those from MGAS, where the numbers of dominant OTUs (>1%) revealed by MGAS were more congruent to those from metagenomics. The improved performance with the MGAS method in characterizing SDP diversity is likely due to greater sequence divergence of the marker genes. For instance, the average pairwise inter-SDPs sequence similarity in the *frr* hyper-variable region was significantly lower (90.92% ± 3.18, n = 65) than that of the 16S V4 region (99.95% ± 0.65, n = 44) (Wilcoxon rank-sum test, *P* < 2 e-16). We reconstructed the *Gilliamella* phylogeny using both *frr* and the 16S V4. The results showed that *frr* revealed a correct SDP relationship, while 16S V4 yielded an intermingled cluster containing SDPs from Gillia_Acer_1 and Gillia_Acer_2, with nearly no in-group resolution (Fig. S7). Furthermore, when blasted against the reference genome data set, 16S V4 almost always failed to assign sequences as the correct SDPs, with only one exception that was mapped to Gillia_Acer_3.

## SUMMARY AND DISCUSSION

We developed a pipeline to identify reliable marker genes for accurate identification and quantification of SDPs from bacterial communities. Three important criteria were applied in the assessment: the extent of sequence divergence, phylogenetic accuracy, and the presence of flanking conservative primer regions. Single-copy protein-coding genes identified by our pipeline were applied as marker genes in SDP quantification of honeybee gut microbiota, successfully producing results consistent with those from metagenomics, which were used as the gold standard. Conversely, we showed that the widely used 16S V4 region contained limited sequence divergence within phylotypes, failing to provide sufficient resolution in differentiating SDPs. As a result, 16S V4 amplicon sequencing cannot reflect fine scale bacterial diversity for the community. Consequently, dominant OTUs delineated by 16S V4 at 97% or 99% thresholds significantly differed from the defined SDPs. On the other hand, the OTUs of single-copy protein-coding genes screened out by our pipeline were successfully assigned to the correct SDPs, and the numbers of dominant OTUs showed more congruent results to those from metagenomics.

Compared with whole-genome shotgun sequencing, amplicon sequencing of single-copy protein-coding genes provides an alternative solution to characterize SDP diversity in an accurate, quantitative and economical way. We address that not every single copy protein-coding gene is efficacious in SDP quantification. The candidate gene must meet all three criteria integrated in our pipeline to be a good marker gene. Notably, the three marker genes identified in this study were screened from a small set of candidates containing only 15 genes recommended for bacterial species identification. We emphasize that these genes are not meant to be exhaustive for bacterial SDP profiling. A larger candidate gene set (e.g., the 114 PhyEco bacterial universal genes reported in Wu et al. [25]) or even the whole set of single-copy genes that are conservative across bacterial lineages) will likely result in a lot more marker genes suitable for identifying and quantifying bacterial SDPs. In this case, we expect dozens to hundreds of proper marker genes to be filtered out. On the other hand, a small set of core single-copy protein-copy genes that are determined to be universally present among known bacteria, such as the 15 marker genes tested in this study, will likely provide candidate genes suitable for accurate characterization of SDP diversity for less known bacterial taxa.

Accurate identification of the SDP composition will also facilitate the prediction of the functional capacity of microbial communities. Functional attributes of a given bacterial lineage are strongly correlated with its phylogenetic position (43). Therefore, various approaches, e.g., PICRUTs (44), have been developed to predict potential functions of a given microbial community based on phylogenetic profiles of bacterial members. As demonstrated in this study, single-copy protein-coding genes identified by our pipeline show better fidelity in revealing phylogenetic relationships for the focal phylotype. Therefore, we anticipate that function prediction for microbial communities will be further improved by integrating single-copy protein-coding genes and the screening pipeline described here.

## MATERIALS AND METHODS

### Genome references of core gut bacteria of honeybees.

A total of 242 bacterial genomes associated with *A. mellifera* and *A. cerana* were downloaded from the NCBI genome database (Table S1). These 242 genomes were used as the reference database of honeybee gut bacteria, which comprised the 6 major phylotypes: *Apibacter* (n = 16), *Bifidobacterium* (n = 28), *Lactobacillus* Firm4 (n = 2), *Lactobacillus* Firm5 (n = 24), *Gilliamella* (n = 130) and *Snodgrassella* (n = 42).

### SDP delineation for honeybee core phylotypes.

Protein-coding genes of all sequenced genomes were annotated using Prokka (https://github.com/tseemann/prokka) (45). Core genes, which were defined as being shared by > 99% strains of a given phylotype, were identified using Roary (version 3.13.0) (46) with the parameter -blastp 75. Multiple sequence alignments were carried out using MAFFT (version v7.467, https://github.com/The-Bioinformatics-Group/Albiorix/wiki/mafft) (47). Phylogenetic trees were constructed using core single-copy genes of each phylotype by RAxML (version 8.2.12, -x 12345 -N 1000 -p 12345 -f a -m GTRGAMMA) (48). Phylogenies were visualized in R (version 3.6.0) using the package ggtree_v2.4.1 (49) or iTOL (version 6.1.1) (50). Pairwise genome-wide average nucleotide identity (gANI) values were calculated using pyani (version 0.2.10; https://github.com/widdowquinn/pyani) (51). A clade with a gANI ≥ 95% from its closest clade was defined as an SDP.

### Screening for candidate marker genes capable of discriminating *Gilliamella* SDPs.

The 15 universal single-copy maker genes (*frr, nusA, pth, rbfA, recR, rnhB, ribF, rimM, rsfS, RuvA, smpB, truB, miaA, murB*, and *yebY*, listed in Table S2) (26) were evaluated as candidate genes. The sequences of candidate marker genes were retrieved by MIDAS (version 1.3.2) (26), whereas the 16S genes were retrieved from the reference genomes using an in-house script. The average Shannon entropy (ASE) of the full gene length was used to assess sequence variation between strains of inter- and intra-SDPs for all phylotypes, where the Shannon entropy for each nucleotide site across genomes in comparison was calculated using oligotyping (version 2.1) (52).

The phylotype *Gilliamella*, which contains the most genomes available for this study, was used as a proof of concept to examine the efficacy of marker genes in SDP differentiation. For each SDPs in phylotype *Gilliamella*, the Shannon entropy values were subsequently averaged for each 20-bp slide-window with a 5-bp step to evaluate the regional genetic divergence along the full length of the marker genes. Pairwise sequence similarities were determined by Clustal Omega (53).

From the candidate genes, potential marker genes that may efficiently distinguish all known SDPs of the *Gilliamella* phylotype were screened. The following criteria were followed: (i) the marker genes should contain conservative regions flanking the hyper-variable region for designing primers enabling recovery target phylotype; (ii) the amplicon length is between ∼150-550 bp; (iii) the amplified region is sufficiently variable to allow the discrimination of SDPs; and (iv) the primers are specific to the focal phylotype to avoid off-target amplifications. The aforementioned 15 marker genes were subject to these criteria, and 5 of them (*ffr*, *nusA*, *pth*, *truB*, and *smpB*) were selected as potential markers for identifying SDPs of *A. cerana Gilliamella*. Among these, three genes (*ffr*, *nusA*, and *pth*) were subjected to further testing as a proof of concept, because their amplicon lengths were 206, 206 and 230 bp, respectively, which were ideal for current shotgun sequencing platforms. To increase the throughput and cost efficiency, 24 amplicons were pooled for one sequencing run. The 5′ end of both forward and reverse primers were tagged with 6-bp unique barcode sequences (see Table S3) to distinguish positive and negative DNA strains, and to differentiate samples.

### Bacterial mock samples.

One representative strain from each of the five *Gilliamella* SDPs associated with *A. cerana* was cultured at 35°C and 5% CO_2_ for 48 h, on heart infusion agar (HIA) medium containing 5% sheep’s blood (54). To screen potential contaminations, the full-length 16S gene was amplified for each bacterial culture using universal primers 27F and 1492R (54) and was subject to Sanger sequencing. 16S sequences were checked against those of the reference strains for identification, before strains were mixed for mock samples. Each *Gilliamella* culture was adjusted to OD600 = 0.5. Twenty-four mock SDP communities were prepared by mixing up 2–5 of the representative strains at varied proportions. The compositions of the mock samples were set as: equal proportion of each of the five strains, equal proportion of four strains with the absence of one strain at a time, equal proportion of three strains with the absence of two randomly selected strains, and a series of varied compositions with relative abundances ranging from ca. 0.02% to 50%. DNA of the bacterial mixtures were extracted using a CTAB-based DNA extraction protocol followed by recovery in 10 mM Tris-EDTA buffer (1×TE, pH 7.4) and quantified using the Qubit DNA assay kit on a Qubit 3.0 Fluorometer (Life Technologies, CA, USA). Alternatively, genomic DNA of each of the five representative strain cultures was extracted separately and the mixed at varied compositions and proportions (see Table S4).

### SDP identification and quantification for mock samples using amplicon sequencing of the three marker genes.

PCR amplification was performed for *frr* (frr-F 5′-GCTGAAGATGCAAGAAC and frr-R 5′-GCATCACGACGAATATT), *nusA* (nusA-F 5′-CTTGAAATTGAAGAACT and nusA-R 5′-GTACCTTGTTCAGCTAA), and *pth* (pTH-F 5′-AAACTTATTGTAGG and pTH-R 5′-CCACTTAAATTCATAAA) for each mock sample with three replicates. Triplicate 50-μl reactions were carried out with 25 μl of 2 × Phanta Max Master Mix (Vazyme Biotech, Nanjing, China), 2 μl (each) of 10 μM primer, 19 μl of ddH_2_O, and 2 μl of template DNA. The thermocycling profile consisted of an initial 3-min denaturation at 95°C, 35 cycles of 15 s at 95°C, 15 s at 52°C for *nusA* and *frr* or at 42°C for *pth* and 20 s at 72°C and a final 10-min extension step at 72°C. After being visualized on 2% agarose gels, DNA was purified using a gel extraction kit (Qiagen, Germany) and quantified using the Qubit DNA assay kit on a Qubit 3.0 Fluorometer. Barcoded amplicons of up to 24 mock samples were pooled and subject to Illumina sequencing using a NovaSeq 6000 platform (PCR-free library, 150 PE) at Novogene (Beijing, China). Approximately 1 Gb of raw data were obtained from each pooled library (Table S5).

The program fastq-multx (version 1.3.1. https://github.com/brwnj/fastq-multx) was employed to demultiplex sequencing reads based on barcode sequences. The 6-bp barcodes in reverse sequences were trimmed using Seqtk (https://github.com/lh3/seqtk). The demultiplexed paired-end reads were then analyzed in QIIME2 (version 2020.2. https://qiime2.org) (55). A plugin DATA2 (56) was used to denoise reads and to group sequences into amplicon sequence variants (ASVs). Individual ASVs were then taxonomically classified using blast (classify-consensus-blast) at a 97% identity threshold (Fig. S3) against the 3 marker genes (*ffr*, *nusA*, and *pth*) derived from the customized bee gut bacterial data set. The relative abundance of each SDP (RA_SDP_) was calculated as: RA_SDP_ = (NR_SDP_)/(NR_Gillia_)*100, where NR_SDP_ represents the number of reads mapped to the focal SDP and NR_Gillia_ represents the number of reads mapped to all *Gilliamella* SDPs. These estimated abundances were then compared to those of the mock samples. The performance of SDP profiling of the 3 marker genes was evaluated on the basis of accuracy, sensitivity and repeatability. Intraclass correlation coefficient (ICC) with a two way random/mixed (ICC[C,1]) model was used to assess the repeatability of this method using SPSS (version 20.1) (57).

Rarefaction curves were plotted using identified SDP numbers against read numbers, which were used to infer the minimum read number required to detect strains at varied proportions. For each sample, ASVs with a depth <100 were filtered out. Rarefaction was performed using QIIME2 with the plugin alpha-rarefaction and a sampling depth of 40,000 reads per sample and default parameters. Minimum read numbers for identifying SDPs with relative abundances of 0.02%, 1% and 20% were chosen manually.

### SDP identification and quantification for *A. cerana* gut microbiota using 16S V4, marker genes and metagenome sequencing.

Adult worker bees collected in Sichuan were used to quantify *Gilliamella* SDP diversity using three different methods (16S V4 region amplicon sequencing, MGAS and metagenomics sequencing). Bees were first cooled at 4°C for 10 min. Then the entire guts were dissected from the abdomen using sterile forceps and DNA was extracted using a CTAB bead-beating protocol described previously (29).

First, the 16S V4 region was amplified for six bee guts from Sichuan and sequenced using an Illumina Hiseq X 10 platform (250–300 bp insert size, 250 PE) at BGI-Shenzhen (Shenzhen, China). Raw reads obtained for each sample were summarized in Table S6. Data quality control was performed using fastp (version 0.13.1, -q 20 -u 10 -w 16) (58). The demultiplexed sequences were denoised and grouped into ASVs using an open reference method VSEARCH (59) embedded in QIIME 2. The taxonomic identification for ASVs was subsequently performed using the naive-Bayesian classifier trained on the BGM-Db, a curated 16S reference database for the classification of honeybee and bumblebee gut bacteria (60). A feature table and ASVs consisting of filtered 16S reads pertaining to *Gilliamella* was constructed. OTU clustering was performed at both 97% and 99% identity thresholds, respectively, using VSEARCH with cluster-features-de-novo method. Additionally, low-abundant OTUs comprising of <100 reads were removed. Taxonomic assignments for OTUs were performed using blast against the BGM-Db with SDP-level taxonomy. OTU composition heatmaps were generated based on relative abundances and visualized in R.

Second, for each sample, the marker genes *frr* and *pth*, which demonstrated the best and worst performances in accuracy and sensitivity, respectively, among the 3 marker genes, were applied following the same pipeline used in the mock samples. ASVs of the six sample from Sichuan were clustered into OTUs and filtered following the above-mentioned 16S V4 pipeline. Taxonomic assignments for OTUs were performed by blast against *frr* sequences derived from the customized bee gut bacterial genome sequence database.

Finally, metagenome sequencing of four bee (B0108, B0120, B0154, and B0174) guts was performed using an Illumina Hiseq X 10 platform (300–400 bp insert size, 150 PE) at BGI-Shenzhen. Additional metagenomes of eight worker bee guts (BioProject PRJNA705951) were download from NCBI (Table S6). The metagenome sequencing was used as the gold standard for *Gilliamella* diversity distributed in the honeybee guts. Shotgun reads mapped to the *A. cerana* genome (GCF_001442555.1) using BWA aln (version 0.7.16a-r1181, -n 1) (61) were identified as host reads and subsequently excluded. We used the ‘run_midas.py species’ script in MIDAS with default parameters to estimate the relative abundances of SDPs for each sample. Finally, the results from MGAS were compared to those from metagenome sequencing to assess the performance of the marker genes.

### Data availability.

Raw data from MGAS, 16S V4 amplicon and metagenomics sequencing have been submitted to NCBI under BioProject PRJNA772085.
