# Supplementary material for: Amplicon Sequencing of Single-Copy Protein-Coding Genes Reveals Accurate Diversity for Sequence-Discrete Microbiome Populations
Source: Microbiol Spectr. 2022 Apr 13;10(2):e02105-21. doi: 10.1128/spectrum.02105-21 (PMC9045262; doi:10.1128/spectrum.02105-21)
Supplement: SUPPLEMENTAL FILE 1 — Fig. S1-S7, Tables S1-S6. Download spectrum.02105-21-s001.pdf, PDF file, 0.9 MB [file spectrum.02105-21-s001.pdf]

Supplemental Material

Amplicon Sequencing of Single-copy Protein-coding Genes Reveals Accurate Diversity for Sequence-discrete Microbiome Populations

Chengfeng Yang, Qinzhi Su, Min Tang, Shiqi Luo, Hao Zheng, Xue Zhang, Xin Zhou

Table of contents

FIG S1 1
FIG S2 2
FIG S3 3
FIG S4 4
FIG S5 5
FIG S6 6
FIG S7 7
TABLE S1 8
TABLE S2 14
TABLE S3 15
TABLE S4 16
TABLE S5 18
TABLE S6 19

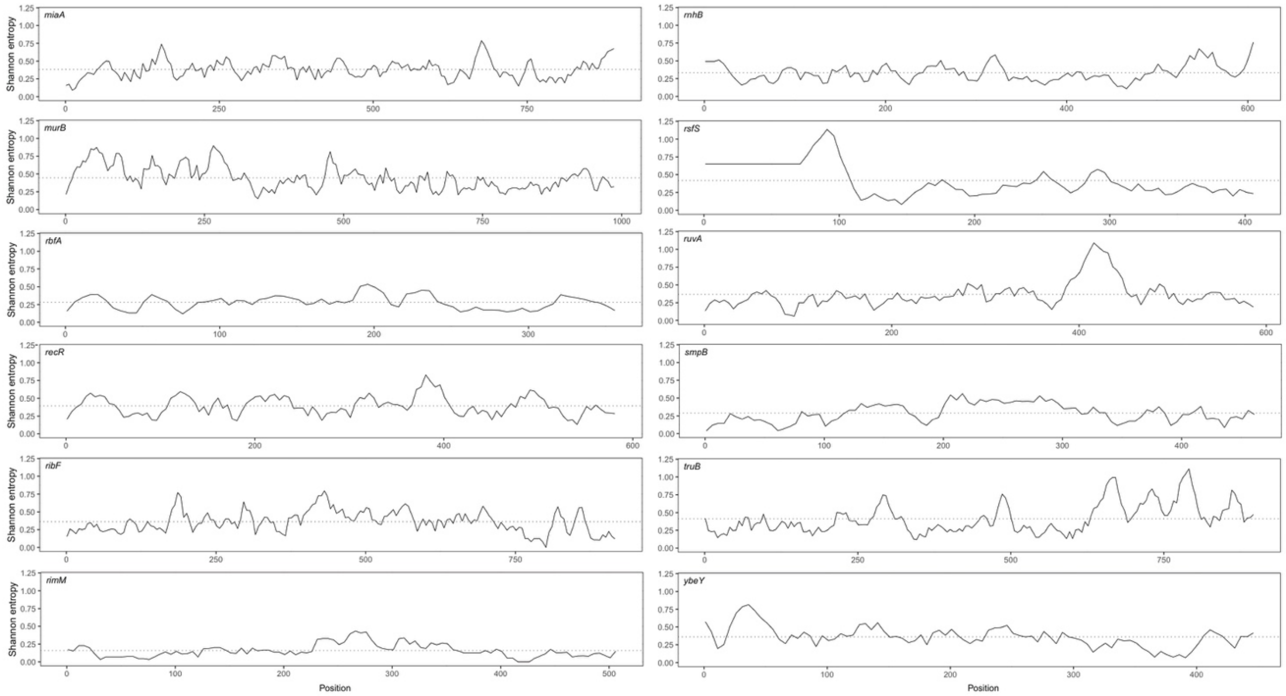

FIG S1 The Shannon entropy across the remain marker genes of all *A. cerana Gilliamella*. The Shannon entropy value is subsequently averaged by a 20-bp slide-window at a 5-bp step. Dash lines represent the mean Shannon entropy values cross all sequences.

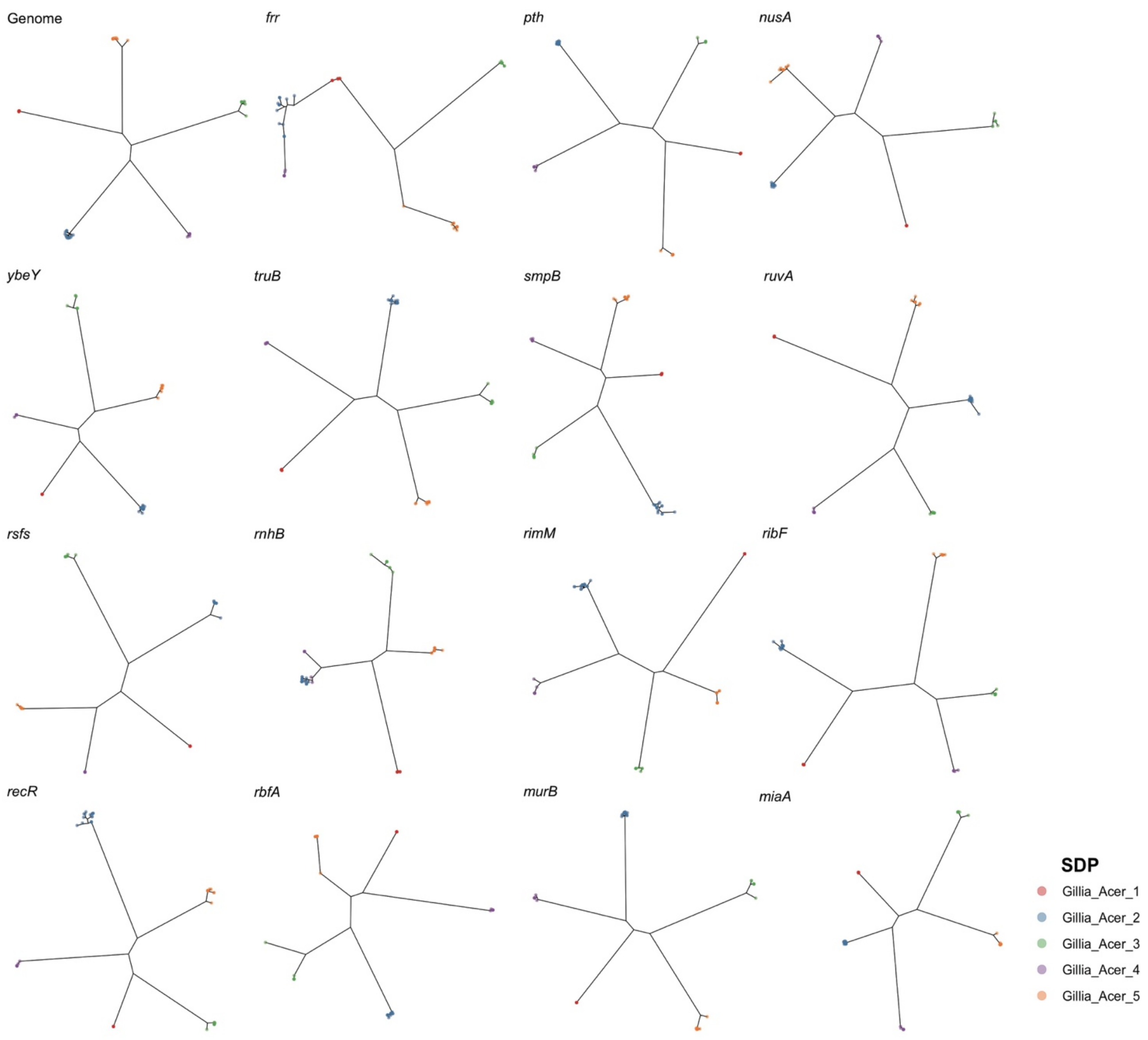

FIG S2 All but *rnhB* of the 15 marker genes produce five SDPs for *A. cerana Gilliamella* phylotype in concert with the whole-genome result.

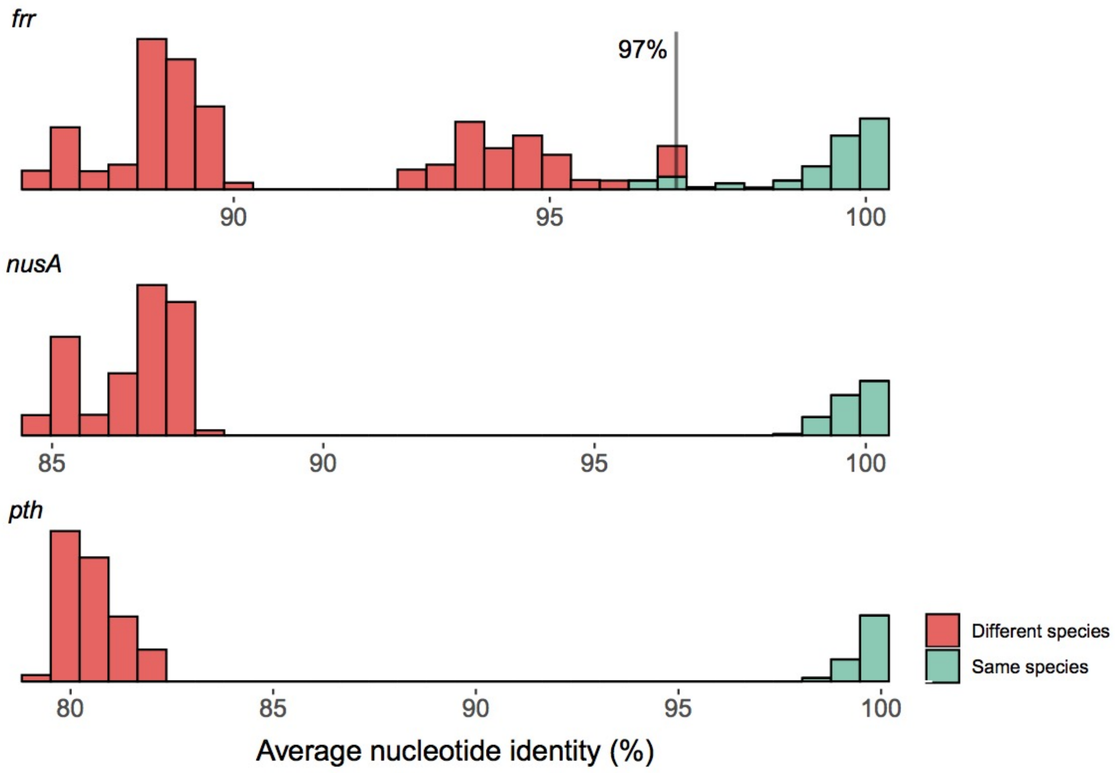

FIG S3 Histograms of average nucleotide identity values of the 3 marker genes from comparisons between strains belonging to the same SDPs (green) or different SDPs (red). Vertical black line indicates the threshold for bacterial SDPs taxonomy for the present method.

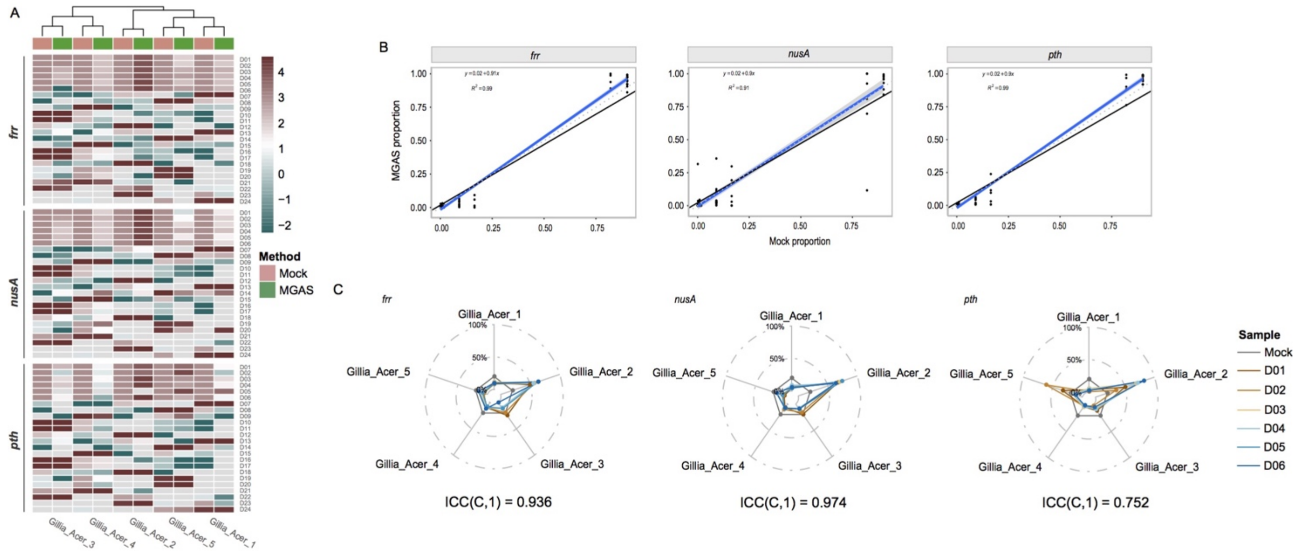

FIG S4 MGAS accurately identifies the *A. cerana Gilliamella* SDPs in DNA mock samples. (A) Relative SDP abundances in mock samples revealed by MGAS. The results shown in the heatmap are the logarithms of the relative abundances percentage of the five representative strains of the five SDPs of *A. cerana Gilliamella*. Grey box indicates a relative abundance at zero. (B) Spearman correlation of SDP abundances in *A. cerana Gillimella* communities revealed by sequencing against mock samples, $p < 2.2e\text{-}16$. The black line presents the linear regression of the MGAS results against SDP abundances in mock samples. The blue solid and gray dashed lines represent a 1: 1 line and the fitted exponential regression (with 95 % confidence interval shown in gray shade), respectively. (C) Repeatability of relative abundance between replicates of DNA mock samples. n = 6, ICC(C,1) is 0.936, 0.974 and 0.752 for *frr*, *nusA* and *pth* genes, respectively.

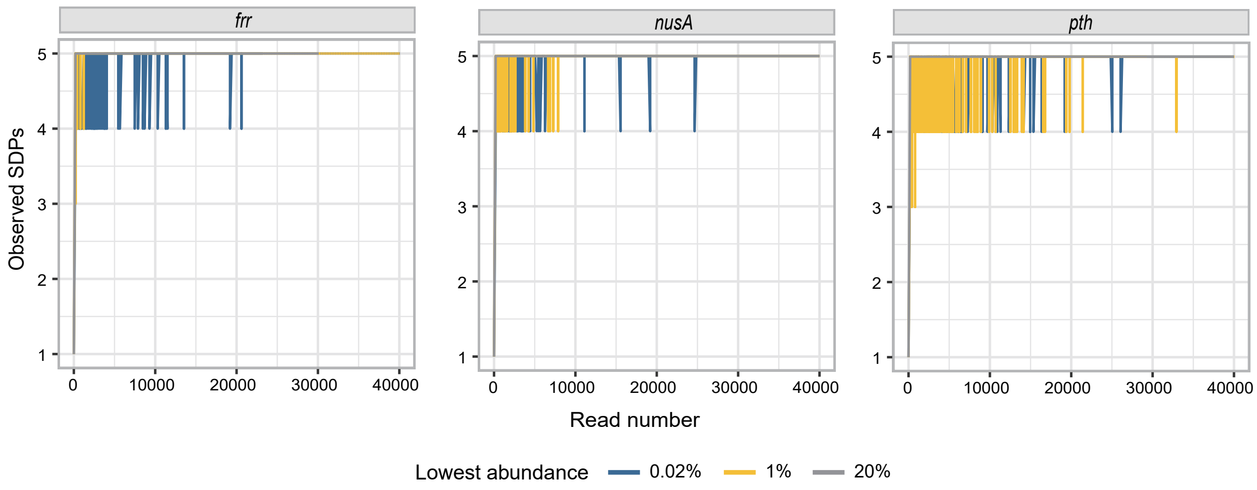

FIG S5 Rarefaction curves of detected bacterial SDPs in bacterial mock samples reach the saturation stage with increasing read numbers.

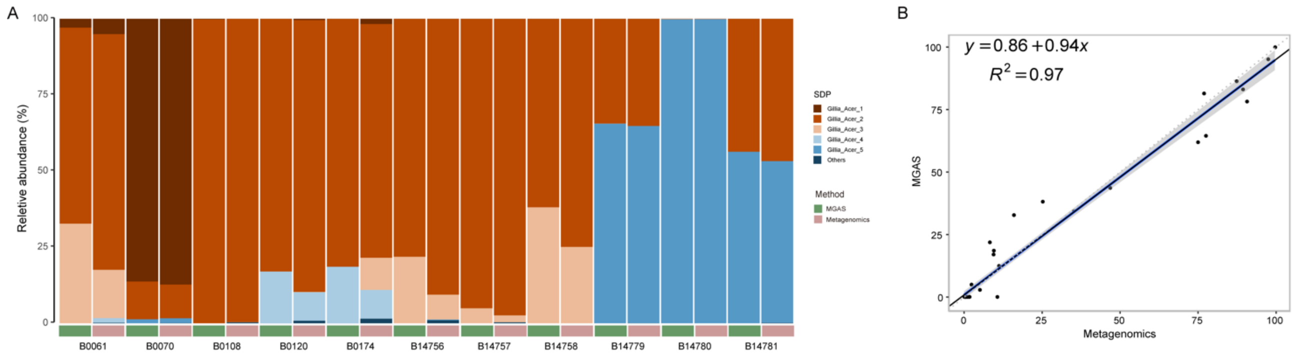

FIG S6 Amplicon sequencing with the *pth* gene showed high congruence to metagenomics sequencing at SDP-level analyses. (A) Relative abundances of *Gilliamella* SDPs revealed by MGAS (*pth* gene) and metagenomics sequencing of *A. cerana* gut communities. (B) Spearman correlation coefficient between MGAS and metagenomics results, with R^2 = 0.97, p < 2.2e-16. The black line presents the linear regression of the MGAS results in SDP abundances against those of metagenomics. The blue solid and gray dashed lines represent a 1: 1 line and the fitted exponential regression (with 95 % confidence interval shown in gray shade), respectively.

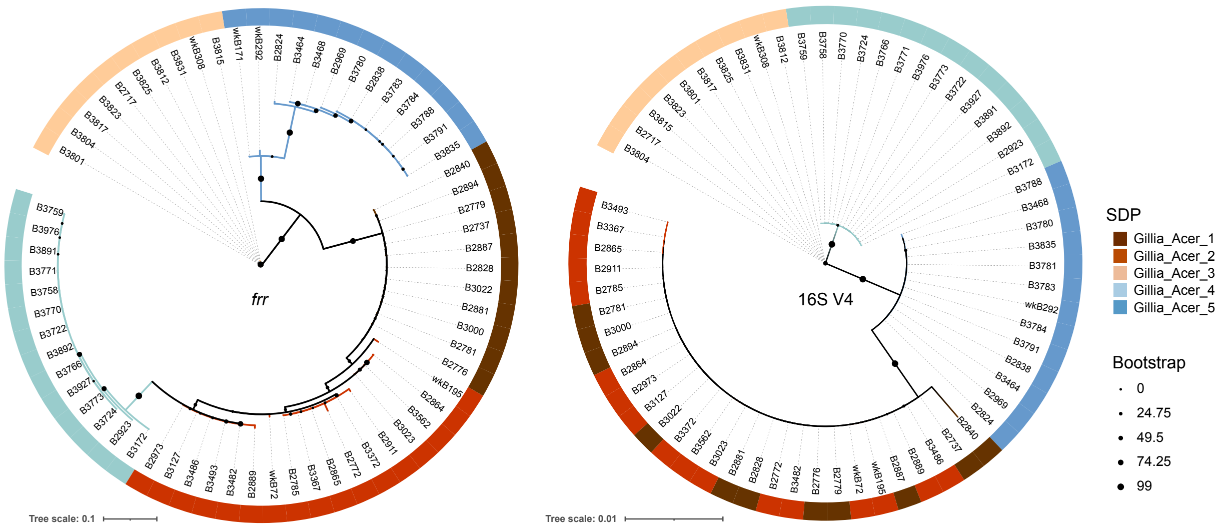

FIG S7 The phylogenetic trees of *A. cerana Gilliamella* built by the amplicon of *frr* (left) or 16S V4 (right).

TABLE S1 Information of the reference genomes

Phylotype	SDP	Strain	Host	Size/Mbp	Contig	Accession
Apibacter	Apibacter_Acer	B2912	*Apis cerana*	2.301246	18	WINV00000000
Apibacter	Apibacter_Acer	B2966	*Apis cerana*	2.315326	Complete	CP049714
Apibacter	Apibacter_Acer	B3239	*Apis cerana*	2.274388	13	WINU00000000
Apibacter	Apibacter_Acer	B3546	*Apis cerana*	2.26779	28	WINW00000000
Apibacter	Apibacter_Acer	B3813	*Apis cerana*	2.327497	14	WINT00000000
Apibacter	Apibacter_Acer	B3883	*Apis cerana*	2.256003	9	WINS00000000
Apibacter	Apibacter_Acer	B3887	*Apis cerana*	2.253703	12	WINR00000000
Apibacter	Apibacter_Acer	B3889	*Apis cerana*	2.256215	8	WINQ00000000
Apibacter	Apibacter_Acer	B3912	*Apis cerana*	2.327696	12	WINP00000000
Apibacter	Apibacter_Acer	B3913	*Apis cerana*	2.328065	14	WINO00000000
Apibacter	Apibacter_Acer	B3918	*Apis cerana*	2.326409	16	WINN00000000
Apibacter	Apibacter_Acer	B3919	*Apis cerana*	2.318874	10	JAFNMG000000000
Apibacter	Apibacter_Acer	B3924	*Apis cerana*	2.323676	20	WINM00000000
Apibacter	Apibacter_Acer	B3935	*Apis cerana*	2.256231	8	WINL00000000
Apibacter	Apibacter_Acer	wkB309	*Apis cerana*	2.29	22	NZ_PSZL00000000
Apibacter	Apibacter_Acer	B3706	*Apis cerana*	2.29768	Complete	CP049715
Bifidobacterium	Bifido_Acer_1	B4079	*Apis cerana*	2.228667	14	JAFNMH000000000
Bifidobacterium	Bifido_Acer_1	B4001	*Apis cerana*	2.228377	14	JAFNMI000000000
Bifidobacterium	Bifido_Acer_2	B4142	*Apis cerana*	2.224927	19	JAFNMJ000000000
Bifidobacterium	Bifido_Acer_2	B3998	*Apis cerana*	2.261594	35	JAFNMK000000000
Bifidobacterium	Bifido_Acer_2	B4077	*Apis cerana*	2.265536	17	JAFNML000000000
Bifidobacterium	Bifido_Acer_2	B4081	*Apis cerana*	2.26482	14	JAFNMM000000000
Bifidobacterium	Bifido_Acer_2	B4107	*Apis cerana*	2.316846	22	JAFNMN000000000
Bifidobacterium	Bifido_Acer_2	B4111	*Apis cerana*	2.316074	21	JAFNMO000000000
Bifidobacterium	Bifido_Acer_2	B4114	*Apis cerana*	2.316582	23	JAFNMP000000000
Bifidobacterium	Bifido_Amel_1	Bin2	*Apis mellifera*	2.09	18	KQ033859
Bifidobacterium	Bifido_Amel_1	1460B	*honeybee*	2.12	38	NZ_PCHJ00000000
Bifidobacterium	Bifido_Amel_2	Bin7	*Apis mellifera*	2.12	11	KQ033885
Bifidobacterium	Bifido_Amel_3	DSM 20089	*Apis mellifera*	2.14	115	JDTU00000000
Bifidobacterium	Bifido_Amel_3	PRL2011	*Apis mellifera var. ligustica*	2.17	Complete	NC_018720
Bifidobacterium	Bifido_Amel_3	ESL0170	*Apis mellifera*	2.17526	7	NZ_QGLH00000000
Bifidobacterium	Bifido_Amel_4	Hma3	*Apis mellifera*	2.25	16	KQ034040
Bifidobacterium	Bifido_Amel_4	wkB338	*Apis mellifera*	2.19	49	NPOR00000000
Bifidobacterium	Bifido_Amel_4	ESL0198	*Apis mellifera*	2.23561	12	NZ_QGLJ00000000
Bifidobacterium	Bifido_Amel_5	ESL0199	*Apis mellifera*	2.16734	7	NZ_QGLK00000000
Bifidobacterium	Bifido_Amel_6	ESL0200	*Apis mellifera*	1.93342	16	NZ_QGLL00000000
Bifidobacterium	Bifido_Amel_7	Bma6	*Apis mellifera*	1.75	6	JXBX00000000
Bifidobacterium	Bifido_Amel_7	DSM 20216	*Apis mellifera*	1.74	67	JDUF00000000
Bifidobacterium	Bifido_Amel_7	LMG 18911	*Apis mellifera*	1.76	Complete	CP007287
Bifidobacterium	Bifido_Amel_7	ESL0197	*Apis mellifera*	1.71524	6	NZ_QGLI00000000

(Continued Table S1)

Bifidobacterium	Bifido_Amel_7	LMG 11587	*Apis mellifera*	1.73	Complete	CP006018
Bifidobacterium	Bifido_Amel_8	7101	*Apis mellifera*	2.12	19	AWUN00000000
Bifidobacterium	Bifido_Amel_9	A11	*Apis mellifera*	2.18	51	AWUO00000000
Bifidobacterium	Bifido_Amel_9	wkB344	*Apis mellifera*	2.12	27	NPOQ00000000
Gilliamella	Gillia_Acer_1	B3022	*Apis cerana*	2.599312	Complete	CP071867
Gilliamella	Gillia_Acer_1	B2737	*Apis cerana*	2.535027	66	JAFNMQ000000000
Gilliamella	Gillia_Acer_1	B2776	*Apis cerana*	2.551661	41	JAFNMR000000000
Gilliamella	Gillia_Acer_1	B2779	*Apis cerana*	2.550827	40	JAFNMS000000000
Gilliamella	Gillia_Acer_1	B2781	*Apis cerana*	2.550312	41	JAFNMT000000000
Gilliamella	Gillia_Acer_1	B2828	*Apis cerana*	2.50577	36	JAFNMU000000000
Gilliamella	Gillia_Acer_1	B2840	*Apis cerana*	2.503695	35	JAFNMV000000000
Gilliamella	Gillia_Acer_1	B2881	*Apis cerana*	2.48214	49	JAFNMW000000000
Gilliamella	Gillia_Acer_1	B2887	*Apis cerana*	2.493398	33	JAFNMX000000000
Gilliamella	Gillia_Acer_1	B2894	*Apis cerana*	2.522803	43	JAFNMY000000000
Gilliamella	Gillia_Acer_1	B3000	*Apis cerana*	2.484341	41	JAFNMZ000000000
Gilliamella	Gillia_Acer_2	B2911	*Apis cerana*	2.528714	32	JAFNNA000000000
Gilliamella	Gillia_Acer_2	wkB195	*Apis cerana*	2.50	38	LZGP00000000
Gilliamella	Gillia_Acer_2	wkB72	*Apis cerana*	2.57	30	LZEI00000000
Gilliamella	Gillia_Acer_2	B2772	*Apis cerana*	2.415473	11	JAFNNB000000000
Gilliamella	Gillia_Acer_2	B2785	*Apis cerana*	2.51783	26	JAFNNC000000000
Gilliamella	Gillia_Acer_2	B2864	*Apis cerana*	2.562399	27	JAFNND000000000
Gilliamella	Gillia_Acer_2	B2865	*Apis cerana*	2.580268	33	JAFNNE000000000
Gilliamella	Gillia_Acer_2	B2889	*Apis cerana*	2.387567	14	JAFNNF000000000
Gilliamella	Gillia_Acer_2	B2973	*Apis cerana*	2.431252	18	JAFNNG000000000
Gilliamella	Gillia_Acer_2	B3023	*Apis cerana*	2.581291	84	JAFNNH000000000
Gilliamella	Gillia_Acer_2	B3127	*Apis cerana*	2.483422	19	JAFNNI000000000
Gilliamella	Gillia_Acer_2	B3367	*Apis cerana*	2.612039	23	JAFNNJ000000000
Gilliamella	Gillia_Acer_2	B3372	*Apis cerana*	2.385574	62	JAFNNK000000000
Gilliamella	Gillia_Acer_2	B3482	*Apis cerana*	2.525265	11	JAFNNL000000000
Gilliamella	Gillia_Acer_2	B3486	*Apis cerana*	2.482707	18	JAFNNM000000000
Gilliamella	Gillia_Acer_2	B3493	*Apis cerana*	2.47007	53	JAFNNN000000000
Gilliamella	Gillia_Acer_2	B3562	*Apis cerana*	2.561588	28	JAFNNO000000000
Gilliamella	Gillia_Acer_3	B3831	*Apis cerana*	2.543295	38	JAFNNP000000000
Gilliamella	Gillia_Acer_3	wkB308	*Apis cerana*	2.74	46	LZGN00000000
Gilliamella	Gillia_Acer_3	B2717	*Apis cerana*	2.511108	96	JAFNNQ000000000
Gilliamella	Gillia_Acer_3	B3801	*Apis cerana*	2.588164	32	JAFNNR000000000
Gilliamella	Gillia_Acer_3	B3804	*Apis cerana*	2.582334	70	JAFNNS000000000
Gilliamella	Gillia_Acer_3	B3812	*Apis cerana*	2.140976	72	JAFNNT000000000
Gilliamella	Gillia_Acer_3	B3815	*Apis cerana*	2.542837	33	JAFNNU000000000
Gilliamella	Gillia_Acer_3	B3817	*Apis cerana*	2.542125	29	JAFNNV000000000
Gilliamella	Gillia_Acer_3	B3823	*Apis cerana*	2.543669	36	JAFNNW000000000
Gilliamella	Gillia_Acer_3	B3825	*Apis cerana*	2.528558	67	JAFNNX000000000
Gilliamella	Gillia_Acer_4	B3976	*Apis cerana*	2.729098	109	JAFNNY000000000

(Continued Table S1)

Gilliamella	Gillia_Acer_4	B2923	*Apis cerana*	2.739944	60	JAFNNZ000000000
Gilliamella	Gillia_Acer_4	B3172	*Apis cerana*	2.673879	32	JAFNOA000000000
Gilliamella	Gillia_Acer_4	B3722	*Apis cerana*	2.742276	52	JAFNOB000000000
Gilliamella	Gillia_Acer_4	B3724	*Apis cerana*	2.744639	50	JAFNOC000000000
Gilliamella	Gillia_Acer_4	B3758	*Apis cerana*	2.744115	53	JAFNOD000000000
Gilliamella	Gillia_Acer_4	B3759	*Apis cerana*	2.743356	47	JAFNOE000000000
Gilliamella	Gillia_Acer_4	B3766	*Apis cerana*	2.73987	57	JAFNOF000000000
Gilliamella	Gillia_Acer_4	B3770	*Apis cerana*	2.680421	112	JAFNOG000000000
Gilliamella	Gillia_Acer_4	B3771	*Apis cerana*	2.619946	117	JAFNOH000000000
Gilliamella	Gillia_Acer_4	B3773	*Apis cerana*	2.742292	54	JAFNOI000000000
Gilliamella	Gillia_Acer_4	B3891	*Apis cerana*	2.742722	57	JAFNOJ000000000
Gilliamella	Gillia_Acer_4	B3892	*Apis cerana*	2.74151	48	JAFNOK000000000
Gilliamella	Gillia_Acer_4	B3927	*Apis cerana*	2.743671	51	JAFNOL000000000
Gilliamella	Gillia_Acer_5	B3835	*Apis cerana*	2.598764	20	JAFNOM000000000
Gilliamella	Gillia_Acer_5	wkB292	*Apis cerana*	2.74	85	LZGO00000000
Gilliamella	Gillia_Acer_5	B2824	*Apis cerana*	2.614205	45	JAFNON000000000
Gilliamella	Gillia_Acer_5	B2838	*Apis cerana*	2.830641	51	JAFNOO000000000
Gilliamella	Gillia_Acer_5	B2969	*Apis cerana*	2.736783	32	JAFNOP000000000
Gilliamella	Gillia_Acer_5	B3464	*Apis cerana*	2.734988	40	JAFNOQ000000000
Gilliamella	Gillia_Acer_5	B3468	*Apis cerana*	2.735561	44	JAFNOR000000000
Gilliamella	Gillia_Acer_5	B3780	*Apis cerana*	2.583753	60	JAFNOS000000000
Gilliamella	Gillia_Acer_5	B3781	*Apis cerana*	2.286034	37	JAFNOT000000000
Gilliamella	Gillia_Acer_5	B3783	*Apis cerana*	2.598931	19	JAFNOU000000000
Gilliamella	Gillia_Acer_5	B3784	*Apis cerana*	2.597354	23	JAFNOV000000000
Gilliamella	Gillia_Acer_5	B3788	*Apis cerana*	2.59909	19	JAFNOW000000000
Gilliamella	Gillia_Acer_5	B3791	*Apis cerana*	2.598524	21	JAFNOX000000000
Gilliamella	Gillia_Amel_1	A-1-24	*Apis mellifera*	3.14	157	MZNE00000000
Gilliamella	Gillia_Amel_1	A-2-24	*Apis mellifera*	3.14	129	MZNF00000000
Gilliamella	Gillia_Amel_1	A-7-12	*Apis mellifera*	3.14	91	NARP00000000
Gilliamella	Gillia_Amel_1	A8	*Apis mellifera*	3.05	226	MZNG00000000
Gilliamella	Gillia_Amel_1	A9	*Apis mellifera*	3.03	362	NARS00000000
Gilliamella	Gillia_Amel_1	A-9-12	*Apis mellifera*	3.15	189	NART00000000
Gilliamella	Gillia_Amel_1	AW13	*Apis mellifera*	3.13	371	NARU00000000
Gilliamella	Gillia_Amel_1	DSM 104097	*Apis mellifera*	3.07705	53	NZ_QICT00000000
Gilliamella	Gillia_Amel_1	ESL0178	*Apis mellifera*	2.88566	18	NZ_QGLQ00000000
Gilliamella	Gillia_Amel_1	N-12-12	*Apis mellifera*	3.08	147	NAHT00000000
Gilliamella	Gillia_Amel_1	N-15-12	*Apis mellifera*	3.05	264	NAHX00000000
Gilliamella	Gillia_Amel_1	N-22	*Apis mellifera*	2.97	92	NASB00000000
Gilliamella	Gillia_Amel_1	N-28	*Apis mellifera*	2.98	304	NASC00000000
Gilliamella	Gillia_Amel_1	N-9-4	*Apis mellifera*	3.07	118	NAHW00000000
Gilliamella	Gillia_Amel_1	N-G5	*Apis mellifera*	3.06	99	NASA00000000
Gilliamella	Gillia_Amel_1	NO10	*Apis mellifera*	3.05	398	NAHU00000000
Gilliamella	Gillia_Amel_1	NO5	*Apis mellifera*	3.06	143	NAHV00000000

(Continued Table S1)

Gilliamella	Gillia_Amel_1	NO6	*Apis mellifera*	3.06	129	NAHR00000000
Gilliamella	Gillia_Amel_1	NO8	*Apis mellifera*	3.04	529	NAHS00000000
Gilliamella	Gillia_Amel_1	P46G	*Apis mellifera*	7.42	2779	MCIV00000000
Gilliamella	Gillia_Amel_1	P54G	*Apis mellifera*	3.11	55	LZGJ00000000
Gilliamella	Gillia_Amel_1	wkB1	*Apis mellifera*	3.14	Complete	CP007445
Gilliamella	Gillia_Amel_2	A-12-12	*Apis mellifera*	2.94	102	NARO00000000
Gilliamella	Gillia_Amel_2	A-7-24	*Apis mellifera*	2.94	107	NARQ00000000
Gilliamella	Gillia_Amel_2	A-8-12	*Apis mellifera*	2.93	110	NARR00000000
Gilliamella	Gillia_Amel_2	AM4	*Apis mellifera*	3.06	166	NARY00000000
Gilliamella	Gillia_Amel_2	AM6	*Apis mellifera*	3.00	655	MZNH00000000
Gilliamella	Gillia_Amel_2	AW11	*Apis mellifera*	2.94	328	NARX00000000
Gilliamella	Gillia_Amel_2	Aw-17	*Apis mellifera*	2.93	130	MZNI00000000
Gilliamella	Gillia_Amel_2	B02	*Apis mellifera*	1.81	544	JAIM00000000
Gilliamella	Gillia_Amel_2	N10	*Apis mellifera*	2.90	202	NARV00000000
Gilliamella	Gillia_Amel_2	N2	*Apis mellifera*	2.91	88	NARW00000000
Gilliamella	Gillia_Amel_2	N4	*Apis mellifera*	2.91	127	NARN00000000
Gilliamella	Gillia_Amel_2	N6	*Apis mellifera*	2.91	82	NARZ00000000
Gilliamella	Gillia_Amel_2	wkB7	*Apis mellifera*	2.90	Complete	LZGG01000000
Gilliamella	Gillia_Amel_3	N-G2	*Apis mellifera*	2.71	77	NAST00000000
Gilliamella	Gillia_Amel_3	N-W3	*Apis mellifera*	2.67	90	NASW00000000
Gilliamella	Gillia_Amel_4	A7	*Apis mellifera*	2.71	125	NASN00000000
Gilliamella	Gillia_Amel_4	M6-3G	*Apis mellifera*	2.71	85	MCIU00000000
Gilliamella	Gillia_Amel_4	ESL0172	*Apis mellifera*	2.68577	17	NZ_QGLO00000000
Gilliamella	Gillia_Amel_5	A-4-12	*Apis mellifera*	2.63	104	NASK00000000
Gilliamella	Gillia_Amel_5	AM1	*Apis mellifera*	2.44	120	NASL00000000
Gilliamella	Gillia_Amel_5	A-TSA1	*Apis mellifera*	2.47	79	NASO00000000
Gilliamella	Gillia_Amel_5	A-TSA2	*Apis mellifera*	2.46	72	NASP00000000
Gilliamella	Gillia_Amel_5	A-TSA3	*Apis mellifera*	2.47	77	NASQ00000000
Gilliamella	Gillia_Amel_5	A-TSA4	*Apis mellifera*	2.46	86	NASR00000000
Gilliamella	Gillia_Amel_5	ESL0169	*Apis mellifera*	2.43078	13	NZ_QGLN00000000
Gilliamella	Gillia_Amel_5	M1-2G	*Apis mellifera*	2.39	10	LZGQ00000000
Gilliamella	Gillia_Amel_5	N-G1	*Apis mellifera*	2.48	65	NASS00000000
Gilliamella	Gillia_Amel_5	N-G3	*Apis mellifera*	2.48	83	NASU00000000
Gilliamella	Gillia_Amel_5	N-G4	*Apis mellifera*	2.44	142	NASV00000000
Gilliamella	Gillia_Amel_5	NO1	*Apis mellifera*	2.52	156	NASI00000000
Gilliamella	Gillia_Amel_5	NO12	*Apis mellifera*	2.53	68	NASH00000000
Gilliamella	Gillia_Amel_5	NO13	*Apis mellifera*	2.53	60	NASG00000000
Gilliamella	Gillia_Amel_5	NO14	*Apis mellifera*	2.52	75	NASF00000000
Gilliamella	Gillia_Amel_5	NO15	*Apis mellifera*	2.52	50	NASJ00000000
Gilliamella	Gillia_Amel_5	NO16	*Apis mellifera*	2.53	57	NASE00000000
Gilliamella	Gillia_Amel_5	NO3	*Apis mellifera*	2.52	54	NASD00000000
Gilliamella	Gillia_Amel_5	NO4	*Apis mellifera*	2.52	214	NASM00000000
Gilliamella	Gillia_Amel_5	P17	*Apis mellifera*	1.47	296	JAIO00000000

(Continued Table S1)

Gilliamella	Gillia_Amel_5	P62G	*Apis mellifera*	2.55	20	LZGI00000000
Gilliamella	Gillia_Amel_5	P83G	*Apis mellifera*	2.49	28	LZGH00000000
Gilliamella	Gillia_Amel_6	ESL0182	*Apis mellifera*	3.53717	31	NZ_QGLR00000000
Gilliamella	Gillia_Amel_6	I20	*Apis mellifera*	2.21	389	JAIN00000000
Gilliamella	Gillia_Amel_6	ESL0177	*Apis mellifera*	3.0862	19	NZ_QGLP00000000
Lactobacillus Firm-4	Lac_Firm4_1	Bin4	*Apis mellifera*	1.82	28	JXJQ00000000
Lactobacillus Firm-4	Lac_Firm4_2	Hon2	*Apis mellifera*	1.81	17	JXBZ00000000
Lactobacillus Firm-5	Lac_Firm5_Acer	B4015	*Apis cerana*	2.37	22	JAFNOY000000000
Lactobacillus Firm-5	Lac_Firm5_Acer	B4005	*Apis cerana*	2.24	16	JAFNOZ000000000
Lactobacillus Firm-5	Lac_Firm5_Acer	B4007	*Apis cerana*	1.88	9	JAFNPA000000000
Lactobacillus Firm-5	Lac_Firm5_Acer	B4010	*Apis cerana*	2.36	42	JAFNPB000000000
Lactobacillus Firm-5	Lac_Firm5_Acer	B4012	*Apis cerana*	2.36	23	JAFNPC000000000
Lactobacillus Firm-5	Lac_Firm5_Acer	B4026	*Apis cerana*	2.19	25	JAFNPD000000000
Lactobacillus Firm-5	Lac_Firm5_Amel_1	ESL0185	*Apis mellifera*	1.68	1	NZ_CP029476
Lactobacillus Firm-5	Lac_Firm5_Amel_1	Hma11	*Apis mellifera*	1.72	16	JXLG00000000
Lactobacillus Firm-5	Lac_Firm5_Amel_1	LMG 26964	*Apis mellifera L.*	1.59	74	NZ_PDKP00000000
Lactobacillus Firm-5	Lac_Firm5_Amel_1	ESL0263	*Apis mellifera*	1.82	12	NZ_REHL00000000
Lactobacillus Firm-5	Lac_Firm5_Amel_1	R-53131	*Apis mellifera*	1.68	19	FMAN00000000
Lactobacillus Firm-5	Lac_Firm5_Amel_2	wkB10	*Apis mellifera*	2.08	32	JRJB00000000
Lactobacillus Firm-5	Lac_Firm5_Amel_3	Hma2	*Apis mellifera*	2.19	40	JXLH00000000
Lactobacillus Firm-5	Lac_Firm5_Amel_4	Biut2	*Apis mellifera*	2.12	37	JXBY00000000
Lactobacillus Firm-5	Lac_Firm5_Amel_4	ESL0186	*Apis mellifera*	2.02	1	NZ_CP029477
Lactobacillus Firm-5	Lac_Firm5_Amel_4	ESL0261	*Apis mellifera*	2.11	17	NZ_REHN00000000
Lactobacillus Firm-5	Lac_Firm5_Amel_5	ESL0184	*Apis mellifera*	2.04	4	NZ_QGLG00000000
Lactobacillus Firm-5	Lac_Firm5_Amel_5	ESL0259	*Apis mellifera*	1.82	18	NZ_REHP00000000
Lactobacillus Firm-5	Lac_Firm5_Amel_5	Hma8	*Apis mellifera*	2.12	23	JXLI00000000
Lactobacillus Firm-5	Lac_Firm5_Amel_5	ESL0260	*Apis mellifera*	1.93	22	NZ_REHO00000000
Lactobacillus Firm-5	Lac_Firm5_Amel_6	Bma5	*Apis mellifera*	2.02	28	JXJR00000000
Lactobacillus Firm-5	Lac_Firm5_Amel_6	ESL0183	*Apis mellifera*	1.87	2	CP029544
Lactobacillus Firm-5	Lac_Firm5_Amel_6	wkB8	*Apis mellifera*	1.93	Complete	CP009531
Lactobacillus Firm-5	Lac_Firm5_Amel_6	ESL0262	*Apis mellifera*	1.87	15	REHM00000000
Snodgrassella	Snod_Acer_1	B3882	*Apis cerana*	2.51	16	JAFNPF000000000
Snodgrassella	Snod_Acer_Aand_Aflo_1	B3088	*Apis cerana*	2.44	39	JAFNPG000000000
Snodgrassella	Snod_Acer_Aand_Aflo_1	B3800	*Apis cerana*	2.23	21	JAFNPH000000000
Snodgrassella	Snod_Acer_Aand_Aflo_1	B3837	*Apis cerana*	2.04	33	JAFNPI000000000
Snodgrassella	Snod_Acer_Aand_Aflo_1	wkB237A	*Apis andreniformis*	2.32	21	MEIM00000000
Snodgrassella	Snod_Acer_Aand_Aflo_1	wkB273	*Apis florea*	2.32	31	MEIL00000000
Snodgrassella	Snod_Acer_Aand_Aflo_1	wkB298B	*Apis cerana*	2.34	42	MEIK00000000
Snodgrassella	Snod_Amel_1	O02	*Apis mellifera*	1.60	259	JAIL00000000
Snodgrassella	Snod_Amel_2	A-10-12	*Apis mellifera*	2.50	63	NAGY01000000
Snodgrassella	Snod_Amel_2	A11	*Apis mellifera*	2.43	122	NAGZ00000000
Snodgrassella	Snod_Amel_2	A-11-12	*Apis mellifera*	2.50	90	NAHA00000000
Snodgrassella	Snod_Amel_2	A-1-12	*Apis mellifera*	2.50	58	NAHB00000000

(Continued Table S1)

Snodgrassella	Snod_Amel_2	A12	*Apis mellifera*	2.40	214	NAGX00000000
Snodgrassella	Snod_Amel_2	A2	*Apis mellifera*	2.43	84	NAHC01000000
Snodgrassella	Snod_Amel_2	A-2-12	*Apis mellifera*	2.50	75	NAHD01000000
Snodgrassella	Snod_Amel_2	A3	*Apis mellifera*	2.43	109	NAHE01000000
Snodgrassella	Snod_Amel_2	A5	*Apis mellifera*	2.43	120	NAHF01000000
Snodgrassella	Snod_Amel_2	A-5-24	*Apis mellifera*	2.49	172	NAHG01000000
Snodgrassella	Snod_Amel_2	A-9-24	*Apis mellifera*	2.50	62	NAHH01000000
Snodgrassella	Snod_Amel_2	Aw-18	*Apis mellifera*	2.50	88	NAGW01000000
Snodgrassella	Snod_Amel_2	Aw-20	*Apis mellifera*	2.50	65	MVDP01000000
Snodgrassella	Snod_Amel_2	E1	*Apis mellifera*	2.39	381	NXEN00000000
Snodgrassella	Snod_Amel_2	ESL0196	*Apis mellifera*	2.45	15	QGLS00000000
Snodgrassella	Snod_Amel_2	J21	*Apis mellifera*	2.33	456	AVQL00000000
Snodgrassella	Snod_Amel_2	MS1-3	*Apis mellifera*	2.50	93	MEIX00000000
Snodgrassella	Snod_Amel_2	N-23	*Apis mellifera*	2.42	128	NAHQ00000000
Snodgrassella	Snod_Amel_2	N9	*Apis mellifera*	2.40	129	NAHK01000000
Snodgrassella	Snod_Amel_2	N-S1	*Apis mellifera*	2.42	98	NAHL00000000
Snodgrassella	Snod_Amel_2	N-S2	*Apis mellifera*	2.42	73	NAHM01000000
Snodgrassella	Snod_Amel_2	N-S4	*Apis mellifera*	2.42	38	NAHO00000000
Snodgrassella	Snod_Amel_2	N-S5	*Apis mellifera*	2.42	77	NAHP00000000
Snodgrassella	Snod_Amel_2	N-W4	*Apis mellifera*	2.42	75	NAHI01000000
Snodgrassella	Snod_Amel_2	N-W7	*Apis mellifera*	2.42	62	NAHJ01000000
Snodgrassella	Snod_Amel_2	O11	*Apis mellifera*	1.37	401	JAIK00000000
Snodgrassella	Snod_Amel_2	P14	*Apis mellifera*	1.31	385	JACG00000000
Snodgrassella	Snod_Amel_2	PEB0171	*Apis mellifera*	2.52	77	MEIV00000000
Snodgrassella	Snod_Amel_2	PEB0178	*Apis mellifera*	2.52	135	MEIW00000000
Snodgrassella	Snod_Amel_2	wkB2	*Apis mellifera*	2.53	Complete	CP007446
Snodgrassella	Snod_Amel_2	N-S3	*Apis mellifera*	2.46	79	NAHN00000000
Snodgrassella	Snod_Amel_2	wkB332	*Apis mellifera*	2.49	30	MEIJ00000000
Snodgrassella	Snod_Amel_2	wkB339	*Apis mellifera*	2.50	27	MEII00000000
Snodgrassella	Snod_Amel_2	wkB9	*Apis mellifera*	2.55	15	MEIN00000000

TABLE S2 Information of the marker genes

PhyEco marker[a]	Gene	Length/bp
B000079	*frr*	558
B000041	*nusA*	1,476
B000103	*pth*	642
B000063	*rbfA*	378
B000080	*recR*	606
B000039	*rnhB*	627
B000096	*ribF*	939
B000086	*rimM*	531
B000062	*rsfS*	315
B000071	*ruvA*	609
B000065	*smpB*	483
B000032	*truB*	921
B000082	*miaA*	912
B000114	*murB*	1,011
B000081	*yebY*	468

[a]Source is from this reference: doi:10.1371/journal.pone.0077033

TABLE S3 List of barcode sequences

Barcode NO.	Forward seq (5'to 3')	Reverse seq (5'to 3')
B01	ATCACG	ACTGAT
B02	CGATGT	ATGAGC
B03	TTAGGC	ATTCCT
B04	TGACCA	CAAAAG
B05	ACAGTG	CAACTA
B06	GCCAAT	CACCGG
B07	CAGATC	CACGAT
B08	ACTTGA	CACTCA
B09	GATCAG	CAGGCG
B10	TAGCTT	CATGGC
B11	GGCTAC	CATTTT
B12	CTTGTA	CCAACA
B13	AGTCAA	CGGAAT
B14	AGTTCC	CTAGCT
B15	ATGTCA	CTATAC
B16	CCGTCC	CTCAGA
B17	GTAGAG	GACGAC
B18	GTCCGC	TAATCG
B19	GTGAAA	TACAGC
B20	GTGGCC	TATAAT
B21	GTTTCG	TCATTC
B22	CGTACG	TCCCGA
B23	GAGTGG	TCGAAG
B24	GGTAGC	TCGGCA

TABLE S4 Mixing ratio of mock samples

SampleID	Mixing ratio of strains[a]					Barcode NO. [b]
	B2776	B2889	B3801	B3172	B3788	
S01	20.00	20.00	20.00	20.00	20.00	B01
S02	25.00	25.00	0.00	25.00	25.00	B02
S03	0.00	25.00	25.00	25.00	25.00	B03
S04	25.00	0.00	25.00	25.00	25.00	B04
S05	25.00	25.00	25.00	0.00	25.00	B05
S06	25.00	25.00	25.00	25.00	0.00	B06
S07	33.33	33.33	0.00	33.33	0.00	B07
S08	0.00	33.33	0.00	33.33	33.33	B08
S09	0.00	0.00	33.33	33.33	33.33	B09
S10	33.33	0.00	33.33	0.00	33.33	B10
S11	33.33	33.33	33.33	0.00	0.00	B11
S12	24.39	24.39	2.44	24.39	24.39	B12
S13	24.94	24.94	0.25	24.94	24.94	B13
S14	24.99	24.99	0.02	24.99	24.99	B14
S15	20.00	20.00	20.00	20.00	20.00	B15
S16	1.00	9.00	50.00	10.00	30.00	B16
S17	50.00	1.00	30.00	9.00	10.00	B17
S18	30.00	50.00	10.00	1.00	9.00	B18
S19	10.00	30.00	9.00	50.00	1.00	B19
S20	9.00	10.00	1.00	30.00	50.00	B20
S21	0.00	0.00	10.00	90.00	0.00	B21
S22	0.00	0.00	1.00	99.00	0.00	B22
S23	0.00	0.00	0.10	99.90	0.00	B23
S24	0.00	0.00	50.00	50.00	0.00	B24
D01	20.00	20.00	20.00	20.00	20.00	B01
D02	20.00	20.00	20.00	20.00	20.00	B02
D03	20.00	20.00	20.00	20.00	20.00	B03
D04	20.00	20.00	20.00	20.00	20.00	B04
D05	20.00	20.00	20.00	20.00	20.00	B05
D06	20.00	20.00	20.00	20.00	20.00	B06
D07	90.00	9.00	0.90	0.09	0.01	B07
D08	9.00	0.90	0.09	0.01	90.00	B08
D09	0.90	0.09	0.01	90.00	9.00	B09
D10	0.09	0.01	90.00	9.00	0.90	B10
D11	0.09	0.01	90.00	9.00	0.90	B11
D12	0.01	90.00	9.00	0.90	0.09	B12
D13	90.00	9.00	0.90	0.09	0.01	B13
D14	9.00	0.90	0.09	0.01	90.00	B14
D15	0.90	0.09	0.01	90.00	9.00	B15
D16	0.09	0.01	90.00	9.00	0.90	B16

(Continued Table S4)

D17	0.09	0.01	90.00	9.00	0.90	B17
D18	0.01	90.00	9.00	0.90	0.09	B18
D19	0.00	0.00	1.64	16.39	81.97	B19
D20	0.00	0.00	1.64	16.39	81.97	B20
D21	0.00	1.64	16.39	81.97	0.00	B21
D22	1.64	16.39	81.97	0.00	0.00	B22
D23	16.39	81.97	0.00	0.00	1.64	B23
D24	81.97	0.00	0.00	1.64	16.39	B24

[a]B2776, B2889, B3801, B3172 and B3788 are the representative strains of Acer_Giliia_1 to Acer_Giliia_5, respectively.
[b]See Table S3.

TABLE S5 Statistics of data outputs

LibraryID[a]	Raw reads	Clean reads	Raw base/G	Clean base/G	Effective rate/%	Q20/%	Q30/%	GC content/%
f1S01-f1S24	2,910,358	2,904,839	0.87	0.87	99.81	98.96	96.80	42.92
f2S01-f2S24	4,370,025	4,362,026	1.31	1.31	99.82	98.99	96.88	42.85
f3S01-f3S24	3,971,727	3,966,181	1.19	1.19	99.86	98.34	94.65	42.84
N1S01-N1S24	3,101,708	3,097,334	0.93	0.93	99.86	98.83	96.33	38.67
N2S01-N2S24	3,455,304	3,451,312	1.04	1.04	99.88	97.96	93.59	38.66
N3S01-N3S24	2,893,355	2,889,594	0.87	0.87	99.87	97.80	93.25	38.64
P1S01-P1S24	5,446,708	5,439,697	1.63	1.63	99.87	99.10	96.48	36.74
P2S01-P2S24	2,698,030	2,694,490	0.81	0.81	99.87	98.97	96.29	36.88
P3S01-P3S24	3,377,356	3,371,139	1.01	1.01	99.82	97.95	93.16	37.08
fD01-fD24	3,599,515	3,595,846	1.08	1.08	99.90	98.41	95.28	42.44
ND01-ND24	4,399,592	4,393,529	1.32	1.32	99.86	98.42	95.02	38.73
PD01-PD24	3,387,737	3,380,942	1.02	1.01	99.80	98.53	95.13	36.64
fB0061-fB14781	5,441,182	5,434,806	1.63	1.63	99.88	99.24	97.36	42.25
PB0061-PB14781	3,591,409	3,587,252	1.08	1.08	99.88	98.80	95.75	36.55

[a]f, N and P represent the *frr*, *nusA* and *pth* gene sequencing; f1-f3, N1-N3 and P1-P3 represent the three replicates; S01-S24 and D01-D24 are mock samples with different ratio of mixing bacterial cells and DNA, respectively, shown in Table S4; BXXXX presents *Apis cerana* gut sample.

TABLE S6 Summary of read processing and data obtained from marker gene, 16S V4 amplicon and metagenomics sequencing of honey bee guts

Gut ID	Raw PE reads[a]				Joined and filtered reads				*Gilliamella* reads	
	16S	*frr*	*pth*	Meta	16S	*frr*	*pth*	Meta	16S	Meta
B0061	84,584	358,851	65,424	33,687,518	82,760	311,842	60,964	5,159,739	25,700	3,185
B0070	85,169	253,390	94,393	37,685,288	83,491	223,179	91,436	5,909,974	47,892	7,661
B0108	83,908	349,570	123,368	45,115,102	82,132	344,248	121,018	4,242,595	39,424	2,189
B0120	85,368	389,432	29,023	34,543,934	83,267	262,273	28,277	4,523,366	22,741	815
B0154	83,691	289,878	-	34,969,488	81,143	226,310	-	4,622,933	26,346	1,086
B0174	84,281	361,728	75,882	38,471,836	81,979	287,357	68,222	6,058,633	18,258	243
B14756	-	224,748	118,334	21,491,068	-	194,850	114,236	4,622,933	-	17,472
B14757	-	354,956	158,879	22,959,909	-	328,188	156,087	4,622,933	-	5,546
B14758	-	277,823	165,638	24,408,709	-	224,100	160,741	9,658,926	-	4,182
B14779	-	342,928	151,481	23,802,654	-	285,197	146,421	7,922,065	-	8,133
B14780	-	301,064	48,088	23,495,452	-	272,069	47,247	7,123,654	-	3,064
B14781	-	291,415	71,187	22,381,871	-	237,037	68,190	9,626,197	-	3,686

[a]16S indicates 16S V4; Meta indicates metagenomics; “-” indicates no test.
